# A mild and efficient synthesis of *N-*aryl glycines by the rearrangement of 2-chloro-*N-*aryl acetamides†

**DOI:** 10.1039/d5ra02497h

**Published:** 2025-06-23

**Authors:** Vamshikrishna Y. Radhakrishna, Khajamohiddin Syed, Vipin A. Nair

**Affiliations:** a Department of Chemistry, REVA University Yelahanka Bangalore Karnataka 560064 India; b Department of Biochemistry and Microbiology, Faculty of Science, Agriculture and Engineering, University of Zululand KwaDlangezwa 3886 South Africa syedk@unizulu.ac.za; c School of Biotechnology, Amrita Vishwa Vidyapeetham, Amritapuri Campus Clappana Kollam Kerala 690525 India vn74nr@gmail.com

## Abstract

A mild and efficient one-pot procedure was developed for the synthesis of substituted *N-*aryl glycines from 2-chloro-*N-*aryl acetamides by intermolecular cyclization in the presence of CuCl_2_·2H_2_O and KOH under reflux condition in acetonitrile medium. The reaction mechanism substantiates the formation of the intermediate 1,4-diarylpiperazine-2,5-dione, which on cleaving with ethanolic KOH afforded the desired products in high yields and in short durations. Both electron-donating and electron-withdrawing substituents on the aromatic rings were well tolerated.

## Abstract 2

An intermolecular cyclization of 2-chloro-*N*-aryl acetamides followed by ring opening of the intermediate 1,4-diarylpiperazine-2,5-diones afforded *N*-aryl glycines in an efficient, one-pot sequential addition process.

## Graphical abstract

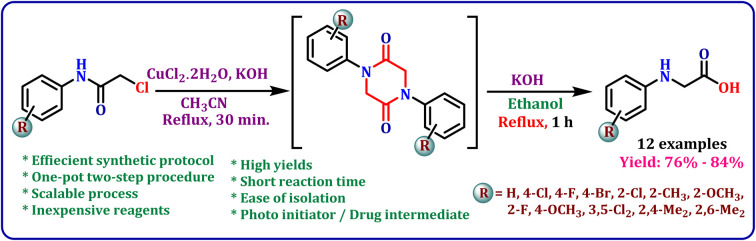

## Introduction

Amino acids constitute one of the most important families of natural products, that play central roles both as structural units of proteins and intermediates in metabolism. Glycine is considered to be an essential amino acid for humans and animals since its shortage can lead to poor immune response, abnormal metabolism of nutrients, retarded growth, and undesirable health effects.^1^ It is implicated in the prevention of many diseases and disorders.^2^ It also finds use as a dietary supplement for enhancing neurological functions.^3–5^*N-*Phenyl glycine (NPG) and its derivatives are known for their biological properties and low toxicity. For example, glycine derivatives of ritodrine exhibited β_3_-adrenoceptor agonistic activity, and their potential was explored for treating frequent urination and urinary incontinence.^6^ In the bacterial biosynthetic pathway, the activated glycine on a carrier protein reacts with diiron oxygenase to give a nitronate intermediate which would be transformed by methyltransferase to form a constituent of the natural product enteromycin carboxamide, an antibacterial agent.^7^

**Fig. 1:**
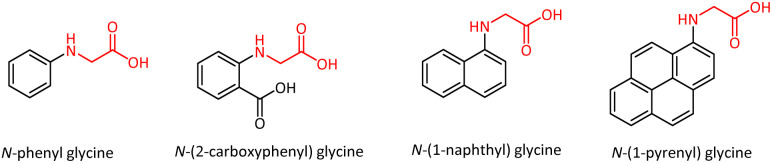
A few *N-*aryl glycines as photoinitiators.

The *N-*aryl glycines have good thermal stability and have garnered considerable interest due to their applications as photopolymerization initiators.^8,9^*N-*Phenyl glycine serves as an initiator for the free radical photopolymerization of acylates and cationic photopolymerization of epoxides and divinyl ethers.^10^ These molecules also made profound impacts in the field of stereolithography and polymeric dental formulations.^11^ Photopolymerization initiators based on *N-*aryl glycines, such as *N-*(1-naphthyl) glycine, *N-*(1-pyrenyl) glycine, *N-*(2-carboxyphenyl) glycine, and *N-*phenyl glycine were developed (Fig. 1).^12^ The *N-*substituted glycine peptoid oligomers were studied as biomimetic foldamers, and the energetic preference for *trans*-amide bond conformers was demonstrated.^13^ BINOL-bearing hexahydropyrrolo[1,2-*c*]imidazol-1-one was identified as an efficient enantioselective fluorescent sensor for the recognition of *N-*Cbz-protected phenyl glycine.^14^

Significant strides were achieved in the synthetic reactions of *N-*aryl glycines. In the presence of a chiral phosphoric acid catalyst and dicyanopyrazine-derived chromophore photosensitizer, enantioselectivity has been achieved in the radical coupling process of substituted *N-*aryl glycines with 1,2-ketones or isatins to form 1,2-amino tertiary alcohols.^15^ Substituted dihydroquinolin-3-ones were obtained from *N-*aryl glycine esters and styrene derivatives by photoinduced tandem Povarov cyclization-oxygenation reaction under metal-free conditions.^16^ In the presence of the catalytic amount of Cu(OTf)_2_, visible light promoted cross-dehydrogenative coupling reaction between imidazo[1,2-*a*]pyridines and *N-*aryl glycine esters.^17^

The decarboxylative coupling reaction of *N-*phenyl glycine with imidazole-fused heterocycles was successful in the presence of CsPbBr_3_ by irradiation with visible light.^18^ Under oxygen atmosphere, the coupling reactions between *N-*aryl glycine esters and acylated amines were carried out with a copper catalyst.^19^ The photoredox Mannich-type reaction between *N-*aryl glycine and silyl enol ether was catalyzed by the photoredox catalyst 5-amino fluorescein and InBr_3_ under visible light.^20^ Radical mediated α-C-H alkylation of glycine provides access to unnatural α-amino acids, which are important intermediates for natural products and biologically active molecules.^21^ In the photoredox cross-dehydrogenative coupling of *N-*aryl glycines with indoles, mesoporous graphitic carbon nitride was employed as a heterogeneous organocatalyst.^22^ Chromeno[2,3*-b*]pyrrol-4(1*H*)-ones were obtained by decarboxylative annulation of 3-formyl chromones with *N-*substituted glycine derivatives using 2-di-*tert*-butyl peroxide and CuBr as the catalyst.^23^*N-*Phenyl glycine provides the methyleneamino synthon through fluorescein-catalyzed decarboxylation, which can undergo an α-amino–alkylation reaction.^24^

**Scheme 1:**
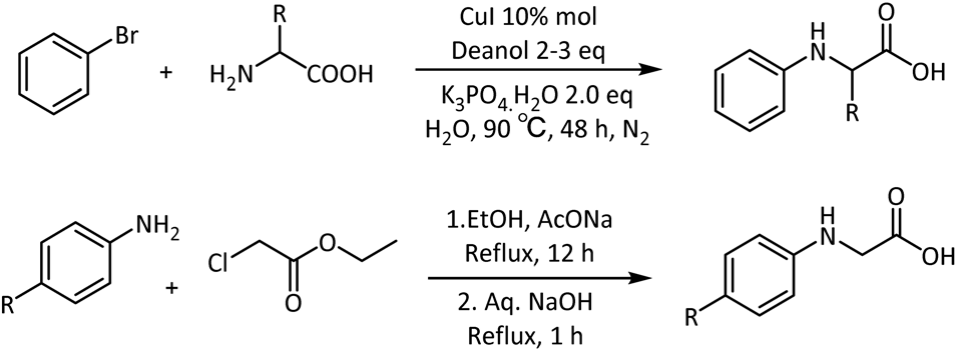
Reported synthetic strategies for *N-*aryl glycines.

The three-component reaction using aldehydes, amines, and cyanide allows the synthesis of amino acids by the Strecker reaction, while the Petasis reaction involves the condensation of organoboronic acids or esters with amines and glyoxylic acids.^25^ One of the strategies to synthesize *N-*alkyl glycine was by the reaction of glycine with benzaldehyde under basic conditions in anhydrous methanol medium, and subsequently reducing the intermediate using sodium borohydride at low temperature.^26^ Another strategy for the *N-*aryl glycine was the conversion of aniline to an aryl/alkyl amine with an ester tethered to the alkyl group. This was achieved in a polar protic medium by reacting aniline and ethyl chloroacetate in the presence of sodium acetate and then hydrolysis of the intermediate to the corresponding amino acid^8^ (Scheme 1). A direct *N-*(hetero)arylation of amino acids was developed in the presence of copper(i) iodide as a catalyst, 2-isobutyryl cyclohexanone as a ligand, and PEG-400 as an additive under MW conditions.^27^ The synthesis of radiolabelled *N-*phenyl glycine was reported by the oxidation of toluene, followed by cyanation with K^14^CN, acid hydrolysis, then bromination at the α-position, and subsequent treatment with liquid ammonia.^28^ With dismay, we realized that all of the above reactions, though afforded the *N-*aryl glycines, were time-consuming, especially with the presence of electron-withdrawing groups on the aromatic amine, often disadvantageous with sluggish reaction rates and moderate overall yields.

## Results and discussion

**Scheme 2:**
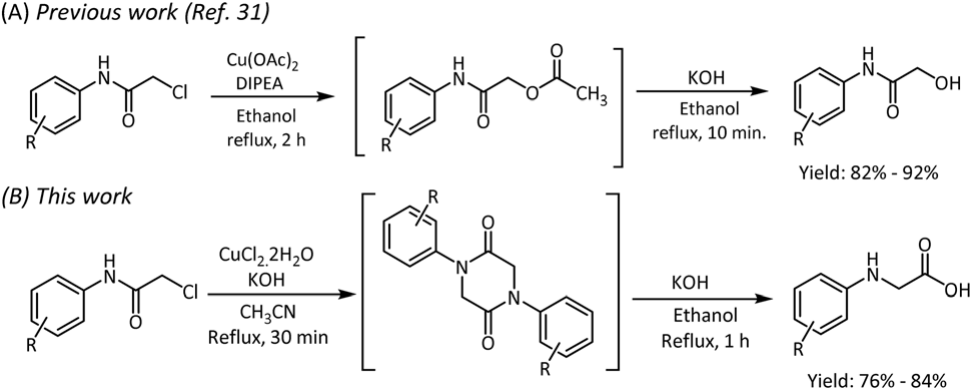
Copper-mediated transformation of 2-chloro-*N*-phenyl acetamide to 2-hydroxy-*N*-phenyl acetamide and *N*-aryl glycine.

Based on the above facts, we decided to explore an economical and efficient process for synthesizing *N-*phenyl glycine by rearranging 2-chloro-*N-*phenyl acetamide through the 1,4-diphenylpiperazine-2,5-dione intermediate. To our curiosity, the literature survey revealed that the reaction of 2-chloro-*N-*phenyl acetamide carried out with the base NaH in THF medium over a temperature range of 0 °C to room temperature yielded 1,4-diphenylpiperazine-2,5-dione in 17 hours.^29^ Under reflux conditions, with NaOH as the base, the reaction time was reduced to 6 hours, however with poor yields.^30^ Considering the drawbacks of these procedures such as poor yield and long reaction time, our efforts were directed to obtain 1,4-diphenylpiperazine-2,5-dione for the synthesis of *N-*phenyl glycine by using metal salts in the presence of a base. Our research group had previously demonstrated the formation of 2-oxo-2-(phenylamino)ethyl acetate intermediate instead of 1,4-diphenylpiperazine-2,5-dione from 2-chloro-*N-*phenyl acetamide by using Cu(OAc)_2_ as an acetate source (Scheme 2). A base-mediated hydrolysis of the ester furnished 2-hydroxy-*N-*phenyl acetamide in good yields within a short duration of time. From this observation, we inferred that Cu(OAc)_2_ is not a suitable Lewis acid for the conversion of 2-chloro-*N-*phenyl acetamide to the piperazine-2,5-dione.^31–33^

Our efforts were therefore directed at identifying a suitable Lewis acid which aids in cyclizing the 2-chloro-*N-*phenyl acetamide to 1,4-diphenylpiperazine-2,5-dione instead of forming the 2-oxo-2-(phenylamino)ethyl acetate. The interest stems from the fact that opening the piperazine-2,5-dione would provide *N-*phenyl glycine, a useful intermediate for many biological and physical applications. The effect of metal salts in promoting the intermolecular cyclization of 2-chloro-*N-*phenyl acetamide was examined (Table 1). Among the different metal salts, Cu(OTf)_2_ and CuCl_2_·2H_2_O were scrutinized for the intermolecular cyclization of the acetamide by carbon–nitrogen bond formation. Though both the Lewis acids afforded impressive results, the inexpensive CuCl_2_·2H_2_O was chosen for further studies. It was found to afford the 1,4-diphenylpiperazine-2,5-dione with a high conversion, and subsequent ring opening in an ethanolic solution of KOH afforded 84% yield of *N-*phenyl glycine.

**Table 1:** Screening of various Lewis acids^a^

Entry	Additive	Time (h)	Yield of NPG (%)
1	Cu(OTf)_2_	0.5	76
2	CuCl_2_·2H_2_O	0.5	84
3	CuCl	3	67
4	CuI	2.5	70
5	ZnCl_2_	5	55
6	NiCl_2_	3.5	62
7	FeCl_3_	3	65
8	CuCl_2_·2H_2_O (0.5 mmol)	2.5	74
9	CuCl_2_·2H_2_O (1.5 mmol)	0.5	85
10	CuCl_2_·2H_2_O (2.0 mmol)	0.5	87

a 2-Chloro-*N-*phenyl acetamide (1.0 mmol), KOH (1.1 mmol), Lewis acid (1.1 mmol), CH_3_CN (10 mL), reflux temp., concentrated the solvent, and added KOH (2.5 mmol), EtOH (10 mL), reflux temp.

**Scheme 3:**
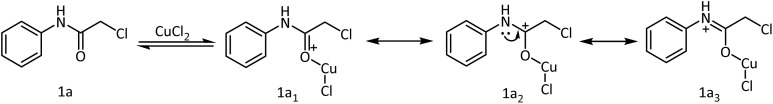
Resonance stabilization of the 2-chloro-*N-*phenyl acetamide-Cu(ii) chloride.

The effectiveness of the Lewis acids originates from their ability to coordinate to electron-rich centers. This facilitates the polarization of the electron density in π-bonds. Consequently, in carbonyl systems, the electron pair drifts towards the more electronegative atom and results in the generation of an adjacent electrophilic center. This center may be subjected to attack by a nucleophile or resonance stabilized by electron donation from an adjacent atom, as is expected by the lone pair of electrons on the nitrogen atom of 2-chloro-*N-*phenyl acetamide to form a metaloxy iminium ion (Scheme 3). Upon base-mediated proton abstraction, the metaloxy imine will be formed. Assisted by the lone pair of electrons from the oxygen, the metaloxy imine would behave as a nitrogen nucleophile to displace the chlorine intermolecularly from the softer electrophilic carbon center of a second molecule. The efficiency of this reaction depends on the coordinating ability of the metal salt. In accordance with the perception, Cu(OTf)_2_ and CuCl_2_·2H_2_O promoted the intermolecular cyclization reaction very efficiently with good yields and low reaction times.

Examination of the metal chlorides of the three transition metals nickel, zinc, and copper indicated the efficiency of Cu(ii) ions in promoting the reaction. This could be rationalized by the feasibility of forming the piperazine-2,5-dione intermediate, identified during the transformation of the 2-chloro-*N-*phenyl acetamide-Cu(ii) complex to the product. The conversion may not be possible with the Ni(ii) and Zn(ii) salts due to their unstable +1 oxidation state and hence poorer results. As expected, the reactions were highly discouraging with the chloride salts of these metal ions.

**Scheme 4:**
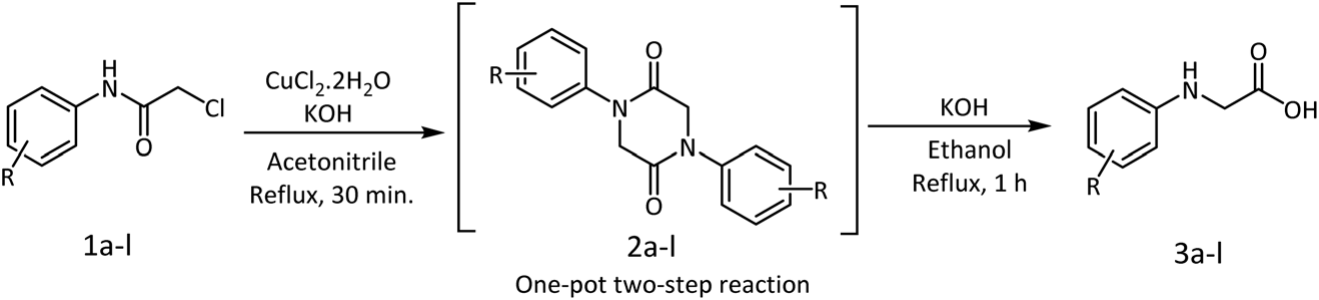
Synthesis of *N-*aryl glycines.

To satisfy our quest on the oxidation state of the metal intermediate, we explored the reaction with the Cu(i) salts, CuCl and CuI. Though the reactions were better than other metal salts, the results were still inferior to CuCl_2_·2H_2_O. The reaction times were considerably high and in particular the yield with CuCl was modest. The longer duration of the reaction may be ascribed to the inefficiency of CuCl to facilitate a high conversion to the piperazine-2,5-dione intermediate, while the propensity was slightly more with CuI. The reaction with FeCl_3_ was found to be inferior even though the reduction of Fe(iii) to form the metaloxy iminium species was envisaged. Thus, CuCl_2_·2H_2_O serves as an excellent Lewis acid by coordinating with the 2-chloro-*N-*phenyl acetamide, thereafter invoking the base-mediated deprotonation and subsequent cyclization to form the piperazine-2,5-dione intermediate (Scheme 4). However, when the reaction was performed with 0.5 mmol of CuCl_2_·2H_2_O low yield was observed even with longer duration of time. Increasing the amount of CuCl_2_·2H_2_O from 1.1 mmol to 1.5 or 2.0 mmol did not improve the yield or reduce the reaction time. Thus the optimization studies indicated the requirement of stoichiometrically equivalent amounts of the starting material and the reagents.

**Scheme 5:**
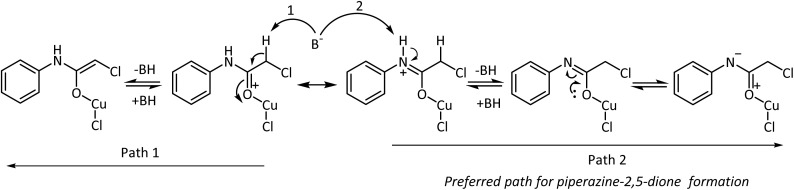
Base-mediated formation of nucleophilic centre.

The role of the base may be examined from the perspective of forming a metaloxy imine intermediate from the corresponding iminium ion, which facilitates the formation of the 1,4-diphenylpiperazine-2,5-dione, and its transformation to *N-*phenyl glycine. Different bases were examined on the model substrate, 2-chloro-*N-*phenyl acetamide (Table 2). Employing sodium acetate in the presence of CuCl_2_·2H_2_O did not significantly alter the progress of the reaction and with NaOMe moderate yields were obtained after a longer duration. The reactions were poor with the organic bases, triethyl amine (TEA) and diisopropyl ethyl amine (DIPEA). The use of the non-nucleophilic base sodium hydride improved the course of the reaction with appreciable yields but the reaction took more time to complete, while NaOH/KOH displayed a better performance in acetonitrile medium in lesser duration. Though the formation of piperazine-2,5-dione using the base K_2_CO_3_ was encouraging, the reaction took a longer duration to complete. The better performance shall be reasoned to the increased conversion of the metaloxy iminium ion to the corresponding imine, and in doing so reduced the possibility of reversal to the starting material (Scheme 5). Upon formation of a resonance stabilized metal coordinated transition state, the course of the reaction will depend upon the ability of the base employed to generate a reasonable concentration of the metaloxy imine to pave the way for 1,4-diphenylpiperazine-2,5-dione formation. This evades the competing reaction of metal enolate formation from 2-chloro-*N-*phenyl acetamide and negates the chances of formation of undesired products. The reaction condition was thus optimized with KOH in acetonitrile medium which gave a better yield in a shorter duration.

**Table 2:** Screening of various bases^a^

Entry	Base	Time (h)	Yield of NPG (%)
1	TEA/DIPEA	24	18
2	NaOAc	24	Trace
3	NaH	9	75
4	NaOH/KOH	0.5	84
5	K_2_CO_3_*	11	82
6	NaOMe	12	25

a 2-Chloro-*N-*phenyl acetamide (1.0 mmol), base (1.1 mmol), CuCl_2_·2H_2_O (1.1 mmol), *EtOH or CH_3_CN (10 mL), reflux temp., concentrated the solvent, and added KOH (2.5 mmol), EtOH (10 mL), reflux temp.

Assuming the existence of charged transition states through which the reaction proceeds, polar solvents would be expected to facilitate the reaction. Therefore, screening of the solvents was done for the transformation of 2-chloro-*N-*phenyl acetamide to the piperazine-2,5-dione (Table 3). The reaction when performed in ethanol medium or ether solvents did not provide good yields. However, with the polar solvents DMSO or DMF, there was a dramatic increase in the rate of the reaction as well as the yields. Satisfactory yields were obtained with the solvent acetonitrile in a shorter duration of time. Temperature was also found to be a significant factor influencing drastically the kinetics of the reaction (Table 4).

**Table 3:** Screening of various solvents^a^

Entry	Solvent	Time (h)	Yield of NPG (%)
1	Acetonitrile	0.5	84
2	Ethanol	12	Trace
3	THF	7	65
4	1,4-Dioxane	5	69
5	DMSO	1	83
6	DMF	1	79

a 2-Chloro-*N-*phenyl acetamide (1.0 mmol), KOH (1.1 mmol), CuCl_2_·2H_2_O (1.1 mmol), solvent (10 mL), reflux temp., concentrated the solvent, and added KOH (2.5 mmol), EtOH (10 mL), reflux temp.

**Table 4:** Influence of temperature^a^

Entry	Temp (^°^C)	Time (h)	Yield of NPG (%)
1	0–5	24	16
2	RT	20	28
3	40	12	58
4	60	2	72
5	80	0.5	84
6	80	1	85
7	80	2	82

a 2-Chloro-*N-*phenyl acetamide (1.0 mmol), KOH (1.1 mmol), CuCl_2_·2H_2_O (1.1 mmol), CH_3_CN (10 mL), heated at the temp. indicated, concentrated the solvent, and added KOH (2.5 mmol), EtOH (10 mL), reflux temp.

With the optimized conditions of temperature and concentrations of CuCl_2·_2H_2_O and KOH, *N-*aryl glycines were synthesized by a one-pot two-step sequential reaction through piperazine-2,5-diones. The intermediate was converted to the desired product in the same reaction flask by the addition of excess ethanolic solution of potassium hydroxide and refluxing. The reactions afforded impressive yields in a short reaction time. Aromatic amines containing the electron donating and withdrawing groups were well tolerated and afforded good yields (Table 5). The scalability of the reaction was examined by converting one gram of 2-chloro-*N-*phenyl acetamide to *N-*phenyl glycine, yielding 84% yield of the product.

**Table 5:** Synthesized *N-*aryl glycine derivatives

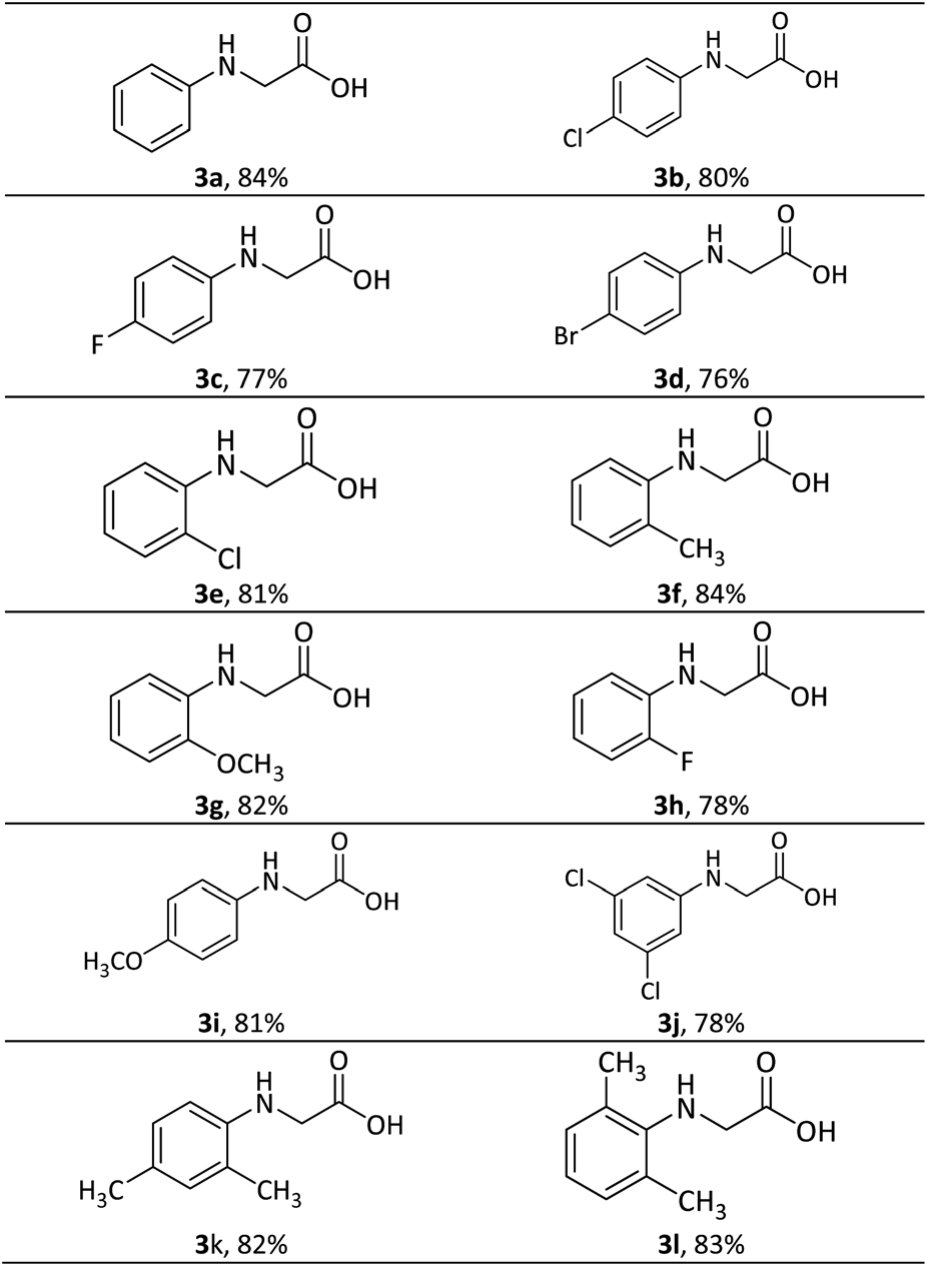

**Scheme 6:**
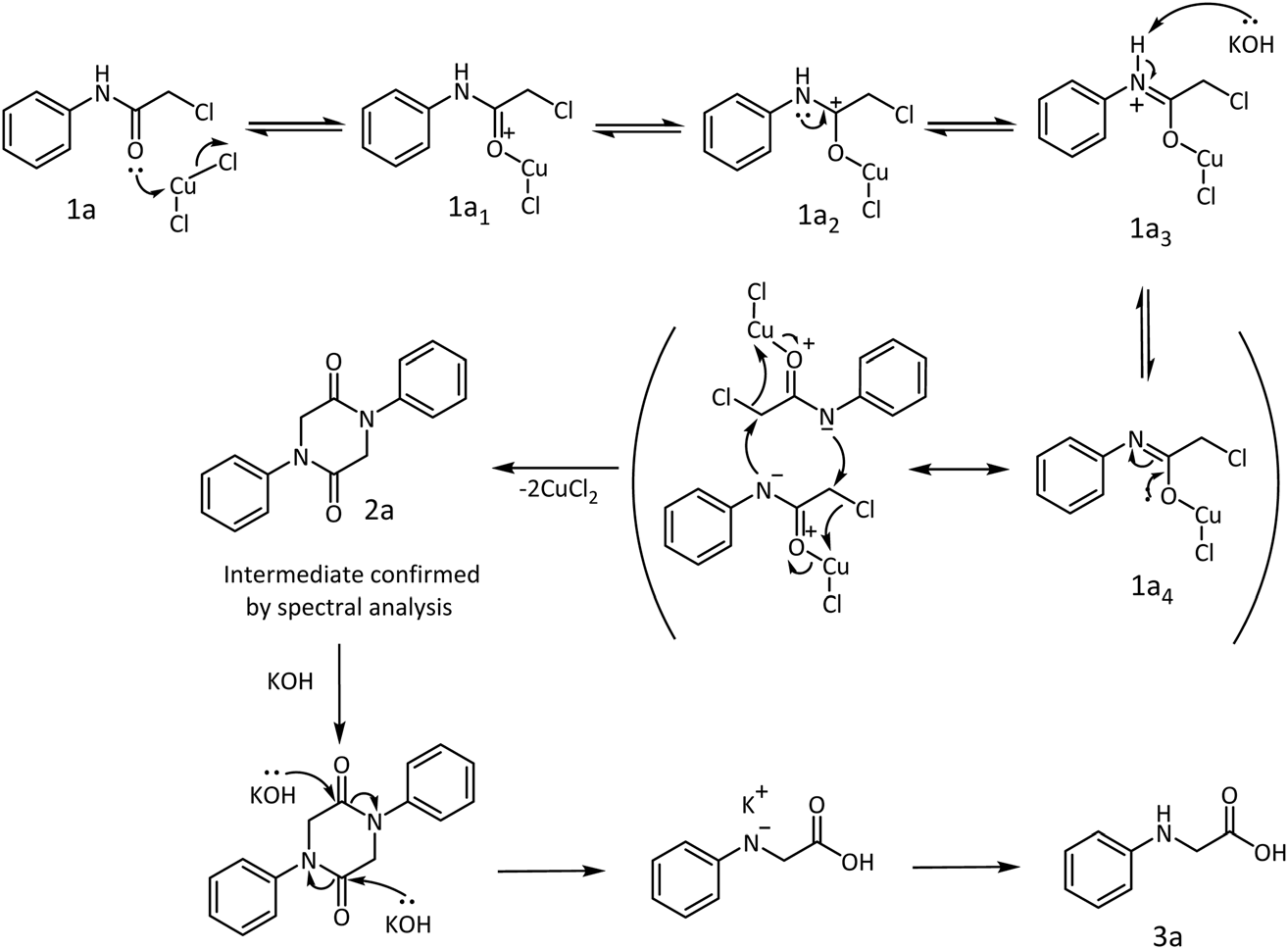
A plausible mechanism for the formation of *N-*phenyl glycine.

A detailed mechanism is proposed to substantiate the product formation. Aniline as the model substrate was treated with chloroacetyl chloride to prepare the starting material 1a for the synthesis of *N-*phenyl glycine. The amide obtained from the reaction was treated with CuCl_2_·2H_2_O. The lone pair of electrons on the carbonyl oxygen would coordinate with the metal salt to form an oxonium ion 1a_1_, which will find stabilization by resonance through the carbonium ion 1a_2_ and iminium ion 1a_3_. Removal of a proton from the iminium ion is facilitated by KOH to yield a metaloxy imine 1a_4_. Assisted by the lone pair of electrons on the oxygen, the metaloxy imine should be sufficiently nucleophilic to attack the chloromethylene side chain intermolecularly and form the 1,4-diphenylpiperazine-2,5-dione 2a intermediate (Scheme 6). The intermediate was identified by NMR spectrometry (ESI†). In an ethanolic alkaline medium, a subsequent ring opening occurs to afford the desired *N-*phenyl glycine 3a. Thus, a two-step one-pot procedure results in the rearrangement of 2-chloro-*N-*phenyl acetamide to *N-*phenyl glycine 3a under the reaction conditions within a short duration.

## Experimental

### General procedure for the preparation of *N*-aryl glycine

To a solution of 2-chloro-*N-*aryl acetamide (1.0 mmol) in acetonitrile (10 mL), KOH (0.06 g, 1.1 mmol) and CuCl_2_·2H_2_O (0.18 g, 1.1 mmol) were added and the resulting mixture was stirred in an oil bath at reflux temperature for 30 minutes. After completion of the reaction as monitored by thin layer chromatography (TLC), the solvent was evaporated under reduced pressure and KOH (0.14 g, 2.5 mmol) in ethanol (10 mL) was added to the reaction mixture and refluxed for another hour. The progress of the reaction was monitored by TLC and upon completion, the reaction mixture was cooled to room temperature and filtered. The filtrate was neutralized by using 2N HCl, diluted with CH_2_Cl_2_ (3 × 15 mL), and washed with water. The combined organic layer was dried over anhydrous Na_2_SO_4_, evaporated under vacuum, and purified by column chromatography on silica gel (60–120 mesh) with ethyl acetate–petroleum ether mixture (30 : 70) as the eluting solvent to obtain the desired product in good yield.

#### *N*-Phenylglycine (3a)

White solid; yield: 0.128 g, 0.85 mmol, 86%; m.p.: 126–128 °C; ^1^H NMR (400 MHz, DMSO-*d*_6_): *δ* 7.34 (d, *J* = 7.9 Hz, 1H, Ar), 7.09 (t, *J* = 7.7 Hz, 2H, Ar), 6.59 (t, *J* = 7.7 Hz, 2H, Ar), 3.80 (s, 2H, CH_2_); ^13^C NMR (100 MHz, DMSO-*d*_6_): *δ* 172.42, 147.22, 128.51, 119.41, 113.52, 44.60; HRMS (ESI): C_8_H_9_NO_2_ [M + H]^+^; calculated: 152.0711; found: 152.0718.

#### *N*-(4-Chlorophenyl)glycine (3b)

White solid; yield: 0.151 g, 0.81 mmol, 82%; m.p.: 144–146 °C; ^1^H NMR (400 MHz, DMSO-*d*_6_): *δ* 7.09 (dd, *J* = 9.3, 3.0 Hz, 2H, Ar), 6.56 (dd, *J* = 9.3, 3.0 Hz, 2H, Ar), 3.78 (s, 2H, CH_2_); ^13^C NMR (100 MHz, DMSO-*d*_6_): *δ* 172.43, 147.21, 128.51, 119.42, 113.52, 44.59; HRMS (ESI): C_8_H_8_ClNO_2_ [M + H]^+^; calculated: 186.0322; found: 186.0329.

#### *N*-(4-Fluorophenyl)glycine (3c)

White solid; yield: 0.135 g, 0.79 mmol, 80%; m.p.: 137–139 °C; ^1^H NMR (400 MHz, DMSO-*d*_6_): *δ* 6.91 (t, *J* = 8.9 Hz, 2H, Ar), 6.53 (dd, *J* = 9.0, 4.5 Hz, 2H, Ar), 3.76 (s, 2H, CH_2_); ^13^C NMR (100 MHz, DMSO-*d*_6_): *δ* 172.65, 155.66, 144.93, 115.08, 112.92, 45.11; HRMS (ESI): C_8_H_8_FNO_2_ [M + H]^+^; calculated: 170.0617; found: 170.0624.

#### *N*-(4-Bromophenyl)glycine (3d)

White solid; yield: 0.183 g, 0.79 mmol, 80%; m.p.: 148–150 °C; ^1^H NMR (400 MHz, DMSO-*d*_6_): *δ* 7.17–7.21 (d, 2H, Ar), 6.49–6.53 (d, 2H, Ar), 3.77 (s, 2H, CH_2_); ^13^C NMR (100 MHz, DMSO-*d*_6_): *δ* 172.38, 147.58, 131.33, 114.10, 106.79, 44.52; HRMS (ESI): C_8_H_8_BrNO_2_ [M + H]^+^; calculated: 229.9816; found: 229.9828.

#### *N*-(2-Chlorophenyl)glycine (3e)

White solid; yield: 0.149 g, 0.85 mmol, 81%; m.p.: 170–172 °C; ^1^H NMR (400 MHz, DMSO-*d*_6_): *δ* 7.27 (s, 1H, NH), 7.22–7.15 (m, 1H, Ar), 7.01–7.08 (m, 1H, Ar), 6.57 (d, *J* = 5.6 Hz, 1H, Ar), 6.44–6.49 (m, 1H, Ar), 3.83 (s, 2H, CH_2_); ^13^C NMR (100 MHz, DMSO-*d*_6_): *δ* 172.30, 143.71, 129.80, 128.14, 117.97, 117.20, 111.53, 44.52; HRMS (ESI): C_8_H_8_ClNO_2_ [M + H]^+^; calculated: 186.0322; found: 186.0327.

#### *N*-(*o*-Tolyl)glycine (3f)

White solid; yield: 0.136 g, 0.82 mmol, 83%; m.p.: 134–136 °C; ^1^H NMR (400 MHz, DMSO-*d*_6_): *δ* 6.98 (t, *J* = 7.7 Hz, 2H, Ar), 6.53 (t, *J* = 7.6 Hz, 1H, Ar), 6.36 (d, *J* = 7.9 Hz, 1H, Ar), 3.83 (s, 2H, CH_2_), 2.09 (s, 3H, CH_3_); ^13^C NMR (100 MHz, DMSO-*d*_6_): *δ* 172.76, 145.85, 129.76, 126.72, 121.64, 116.22, 109.17, 44.87, 17.47; HRMS (ESI): C_9_H_11_NO_2_ [M + H]^+^; calculated: 166.0868; found: 166.0874.

#### *N*-(2-Methoxyphenyl)glycine (3g)

White solid; yield: 0.150 g, 0.82 mmol, 83%; m.p.: 169–171 °C; ^1^H NMR (400 MHz, DMSO-*d*_6_): *δ* 6.82 (d, *J* = 7.9 Hz, 1H, Ar), 6.75 (d, *J* = 7.7 Hz, 1H, Ar), 6.58 (t, *J* = 7.7, 1.4 Hz, 1H, Ar), 6.41 (d, *J* = 7.7 Hz, 1H, Ar), 3.82 (s, 2H, CH_2_), 3.79 (s, 3H, OCH_3_); ^13^C NMR (100 MHz, DMSO-*d*_6_): *δ* 172.53, 146.36, 137.33, 121.00, 116.20, 109.79, 109.38, 55.33, 44.59; HRMS (ESI): C_9_H_11_NO_3_ [M + H]^+^; calculated: 182.0817; found: 182.0827.

#### *N*-(2-Fluorophenyl)glycine (3h)

White solid; yield: 0.135 g, 0.79 mmol, 80%; m.p.: 144–146 °C; ^1^H NMR (400 MHz, DMSO-*d*_6_): *δ* 7.02 (dd, *J* = 12.3, 7.9 Hz, 1H, Ar), 6.95 (t, *J* = 7.7 Hz, 1H, Ar), 6.63–6.53 (m, 2H, Ar), 3.85 (s, 2H, CH_2_); ^13^C NMR (100 MHz, DMSO-*d*_6_): *δ* 172.38, 149.64, 136.34, 124.70, 116.08, 114.27, 112.12, 44.22; HRMS (ESI): C_8_H_8_FNO_2_ [M + H]^+^; calculated: 170.0617; found: 170.0624.

#### *N*-(4-Methoxyphenyl)glycine (3i)

White solid; yield: 0.152 g, 0.83 mmol, 84%; m.p.: 164–166 °C; ^1^H NMR (400 MHz, DMSO-*d*_6_): *δ* 6.71 (d, *J* = 8.7 Hz, 2H, Ar), 6.50 (d, *J* = 8.8 Hz, 2H, Ar), 3.72 (s, 2H, CH_2_), 3.63 (s, 3H, OCH_3_); ^13^C NMR (100 MHz, DMSO-*d*_6_): 172.98, 151.04, 142.44, 114.59, 113.20, 55.36, 45.55; HRMS (ESI): C_9_H_11_NO_3_ [M + H]^+^; calculated: 182.0817; found: 182.0829.

#### *N*-(3,5-Dichlorophenyl)glycine (3j)

White solid; yield: 0.175 g, 0.79 mmol, 80%; m.p.: 174–176 °C; ^1^H NMR (400 MHz, DMSO-*d*_6_): *δ* 12.69 (s, 1H, COOH), 6.63 (s, 1H, NH), 6.57 (s, 2H, Ar), 6.53 (s, 1H, Ar), 3.85 (s, 2H, CH_2_); ^13^C NMR (100 MHz, DMSO-*d*_6_): *δ* 171.96, 150.68, 134.33, 114.79, 110.40, 44.18; HRMS (ESI): C_8_H_7_Cl_2_NO_2_ [M + H]^+^; calculated: 219.9932; found: 219.9942.

#### *N*-(2,4-Dimethylphenyl)glycine (3k)

White solid; yield: 0.150 g, 0.83 mmol, 84%; m.p.: 150–152 °C; ^1^H NMR (400 MHz, DMSO-*d*_6_): *δ* 6.79 (d, *J* = 7.6 Hz, 2H, Ar), 6.26 (d, *J* = 7.9 Hz, 1H, Ar), 3.78 (s, 2H, CH_2_), 2.13 (s, 3H, CH_3_), 2.06 (s, 3H, CH_3_); ^13^C NMR (100 MHz, DMSO-*d*_6_): *δ* 172.91, 143.63, 130.63, 126.95, 124.60, 121.77, 109.38, 45.18, 20.07, 17.43; HRMS (ESI): C_10_H_13_NO_2_ [M + H]^+^; calculated: 180.1024; found: 180.1033.

#### *N*-(2,6-dimethylphenyl)glycine (3l)

White solid; yield: 0.152 g, 0.84 mmol, 85%; m.p.: 152–154 °C; ^1^H NMR (400 MHz, DMSO-*d*_6_): *δ* 7.11 (s, 3H, Ar), 4.08 (s, 2H, CH_2_), 2.38 (s, 6H, 2CH_3_); ^13^C NMR (100 MHz, DMSO-*d*_6_): *δ* 169.54, 136.79, 131.01, 129.61, 126.95, 49.81, 17.88; HRMS (ESI): C_10_H_13_NO_2_ [M + H]^+^; calculated: 180.1024; found: 180.1033.

## Conclusion

An efficient and eco-friendly one-pot two-step procedure was developed for the synthesis of *N-*aryl glycines from 2-chloro-*N-*aryl acetamides using CuCl_2_·2H_2_O and KOH in acetonitrile medium under reflux condition. The reaction proceeds through the formation of 1,4-diarylpiperazine-2,5-dione intermediates, which were converted to the desired products by refluxing in ethanolic KOH solution. The products with electron-donating and withdrawing substituents were obtained in excellent yields in a short duration of time with the ease of isolation.

## Author contributions

V. Y. R., experimental and DATA analysis; K. S., reviewing; V. A. N., conceptualization.

## Conflicts of interest

The authors declare no conflict of interest.

## Supplementary Material

RA-015-D5RA02497H-s001

## Data Availability

The data supporting this article have been included as part of the ESI.†
