# Supplementary material for: A mild and efficient synthesis of N-aryl glycines by the rearrangement of 2-chloro-N-aryl acetamides
Source: RSC Adv. 2025 Jun 23;15(26):21061–7. doi: 10.1039/d5ra02497h (PMC12183544; doi:10.1039/d5ra02497h)
Supplement: RA-015-D5RA02497H-s001 [file RA-015-D5RA02497H-s001.pdf]

Supplementary Information (SI) for RSC Advances.
This journal is © The Royal Society of Chemistry 2025

A mild and efficient synthesis of *N*-aryl glycines by the rearrangement of 2-chloro-*N*-aryl-acetamides

Vamshikrishna Y. Radhakrishna, [a] Khajamohiddin Syed, *[b] and Vipin A. Nair *[c]

[a] *Department of Chemistry, REVA University, Yelahanka, Bangalore, Karnataka 560064, India.*

[b] *Department of Biochemistry and Microbiology, Faculty of Science, Agriculture and Engineering, University of Zululand, KwaDlangezwa 3886, South Africa. Email: syedk@unizulu.ac.za*

[c] *School of Biotechnology, Amrita Vishwa Vidyapeetham, Amritapuri Campus, Clappana, Kollam, Kerala 690525, India. Email: vn74nr@gmail.com.*

Contents	Page No
Figure S1: ^{1}H NMR of *N*-Phenylglycine (3a)	4
Figure S2: ^{13}C NMR of *N*-Phenylglycine (3a)	5
Figure S3: HRMS of *N*-Phenylglycine (3a)	6
Figure S4: ^{1}H NMR of *N*-(4-Chlorophenyl)glycine (3b)	7
Figure S5: ^{13}C NMR of *N*-(4-Chlorophenyl)glycine (3b)	8
Figure S6: HRMS of *N*-(4-Chlorophenyl)glycine (3b)	9
Figure S7: ^{1}H NMR of *N*-(4-Fluorophenyl)glycine (3c)	10
Figure S8: ^{13}C NMR of *N*-(4-Fluorophenyl)glycine (3c)	11
Figure S9: HRMS of *N*-(4-Fluorophenyl)glycine (3c)	12
Figure S10: ^{1}H NMR of *N*-(4-Bromophenyl)glycine (3d)	13
Figure S11: ^{13}C NMR of *N*-(4-Bromophenyl)glycine (3d)	14
Figure S12: HRMS of *N*-(4-Bromophenyl)glycine (3d)	15
Figure S13: ^{1}H NMR of *N*-(2-Chlorophenyl)glycine (3e)	16
Figure S14: ^{13}C NMR of *N*-(2-Chlorophenyl)glycine (3e)	17
Figure S15: HRMS of *N*-(2-Chlorophenyl)glycine (3e)	18
Figure S16: ^{1}H NMR of *N*-(*o*-Tolyl)glycine (3f)	19
Figure S17: ^{13}C NMR of *N*-(*o*-Tolyl)glycine (3f)	20
Figure S18: HRMS of *N*-(*o*-Tolyl)glycine (3f)	21
Figure S19: ^{1}H NMR of *N*-(2-Methoxyphenyl)glycine (3g)	22
Figure S20: ^{13}C NMR of *N*-(2-Methoxyphenyl)glycine (3g)	23
Figure S21: HRMS of *N*-(2-Methoxyphenyl)glycine (3g)	24
Figure S22: ^{1}H NMR of *N*-(2-Fluorophenyl)glycine (3h)	25
Figure S23: ^{13}C NMR of *N*-(2-Fluorophenyl)glycine (3h)	26
Figure S24: HRMS of *N*-(2-Fluorophenyl)glycine (3h)	27

Figure S25: ^{1}H NMR of *N*-(4-Methoxyphenyl)glycine (3i)	28
Figure S26: ^{13}C NMR of *N*-(4-Methoxyphenyl)glycine (3i)	29
Figure S27: HRMS of *N*-(4-Methoxyphenyl)glycine (3i)	30
Figure S28: ^{1}H NMR of *N*-(3,5-Dichlorophenyl)glycine (3j)	31
Figure S29: ^{13}C NMR of *N*-(3,5-Dichlorophenyl)glycine (3j)	32
Figure S30: HRMS of *N*-(3,5-Dichlorophenyl)glycine (3j)	33
Figure S31: ^{1}H NMR of *N*-(2,4-Dimethylphenyl)glycine (3k)	34
Figure S32: ^{13}C NMR of *N*-(2,4-Dimethylphenyl)glycine (3k)	35
Figure S33: HRMS of *N*-(2,4-Dimethylphenyl)glycine (3k)	36
Figure S34: ^{1}H NMR of *N*-(2,6-Dimethylphenyl)glycine (3l)	37
Figure S35: ^{13}C NMR of *N*-(2,6-Dimethylphenyl)glycine (3l)	38
Figure S36: HRMS of *N*-(2,6-Dimethylphenyl)glycine (3l)	39
Figure S37: ^{1}H NMR of 1,4-diphenylpiperazine-2,5-dione (2a)	40
Figure S38: ^{13}C NMR of 1,4-diphenylpiperazine-2,5-dione (2a)	41

Experimental

Materials and Methods

All the chemicals were purchased from commercial suppliers and used as received. The reactions were performed in oven-dried glassware under an appropriate atmosphere. The reaction was monitored by thin-layer chromatography (TLC) on 0.25 mm Merck Silica gel 60 F_{254} plates using UV light for visualization. The column chromatography was performed with 60-120 mesh silica gel using hexane and ethyl acetate as eluents. NMR spectra were recorded on a Jeol ECZ 400R spectrometer (^{1}H at 400 MHz, and ^{13}C at 100 MHz) in DMSO-d_6 as the solvent with TMS as the internal standard. Chemical shifts (δ) are reported relative to residual solvent signals (DMSO-d_6, 2.5 ppm for ^{1}H NMR and septet centered at 39.5 ppm for ^{13}C NMR). Mass spectrometric analysis was carried out in ESI quadrupole time of flight Agilent mass spectrometer.

General procedure for the preparation of N-aryl glycine

To a solution of 2-chloro-*N*-aryl acetamide (1.0 mmol) in acetonitrile, KOH (0.06 g, 1.1 mmol) and $CuCl_2.2H_2O$ (0.18 g, 1.1 mmol) were added, and the resulting mixture was stirred in an oil bath at reflux temperature for 30 minutes. After completion of the reaction as monitored by thin layer chromatography (TLC), the solvent was evaporated under reduced pressure, and KOH (0.14 g, 2.5 mmol) in ethanol was added to the reaction mixture and refluxed for another hour. The progress of the reaction was monitored by TLC, and upon completion the reaction mixture was cooled to room temperature and filtered. The filtrate was neutralized by using 2N HCl, diluted with CH_2Cl_2 (3x15 mL), and washed with water. The combined organic layer was dried over anhydrous Na_2SO_4, evaporated under vacuum, and purified by column chromatography on silica gel (60-120 mesh) with ethyl acetate-petroleum ether mixture (30:70) as the eluting solvent to obtain the desired product in good yields.

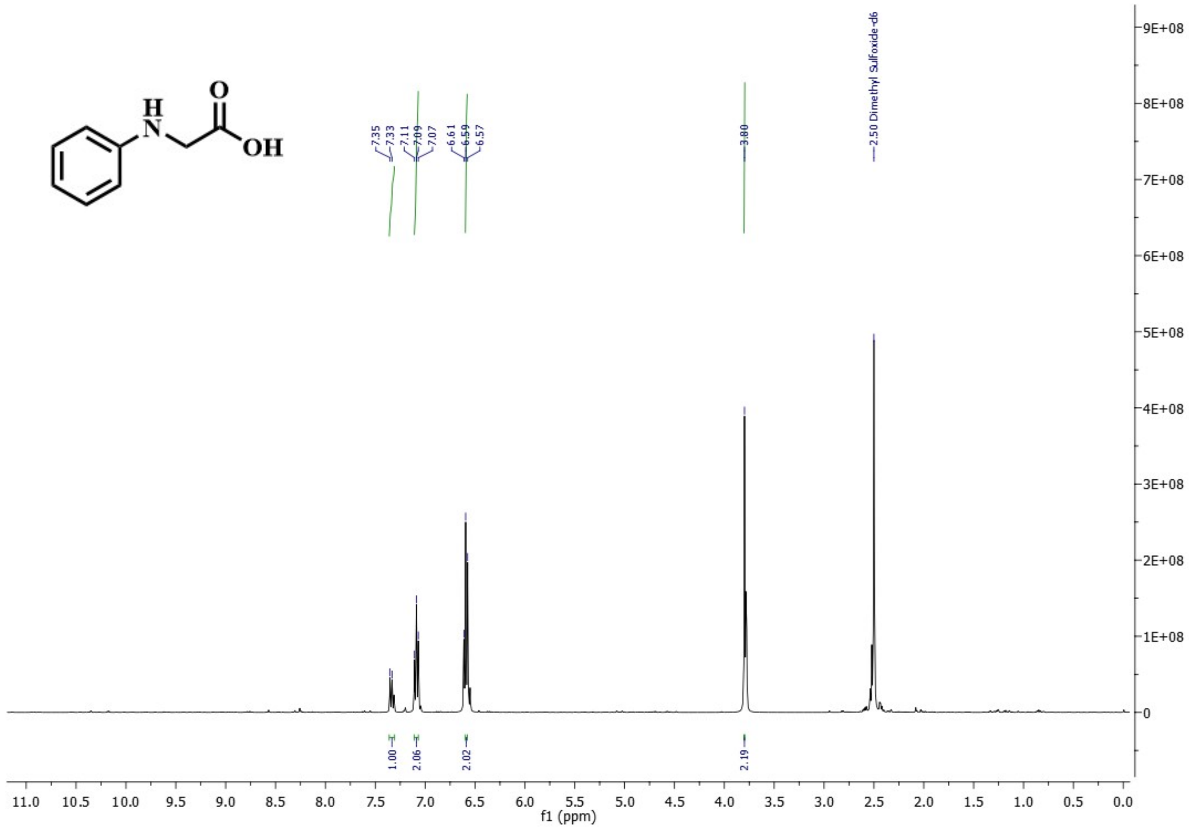

Figure S1: ^{1}H NMR of *N*-Phenylglycine (3a)

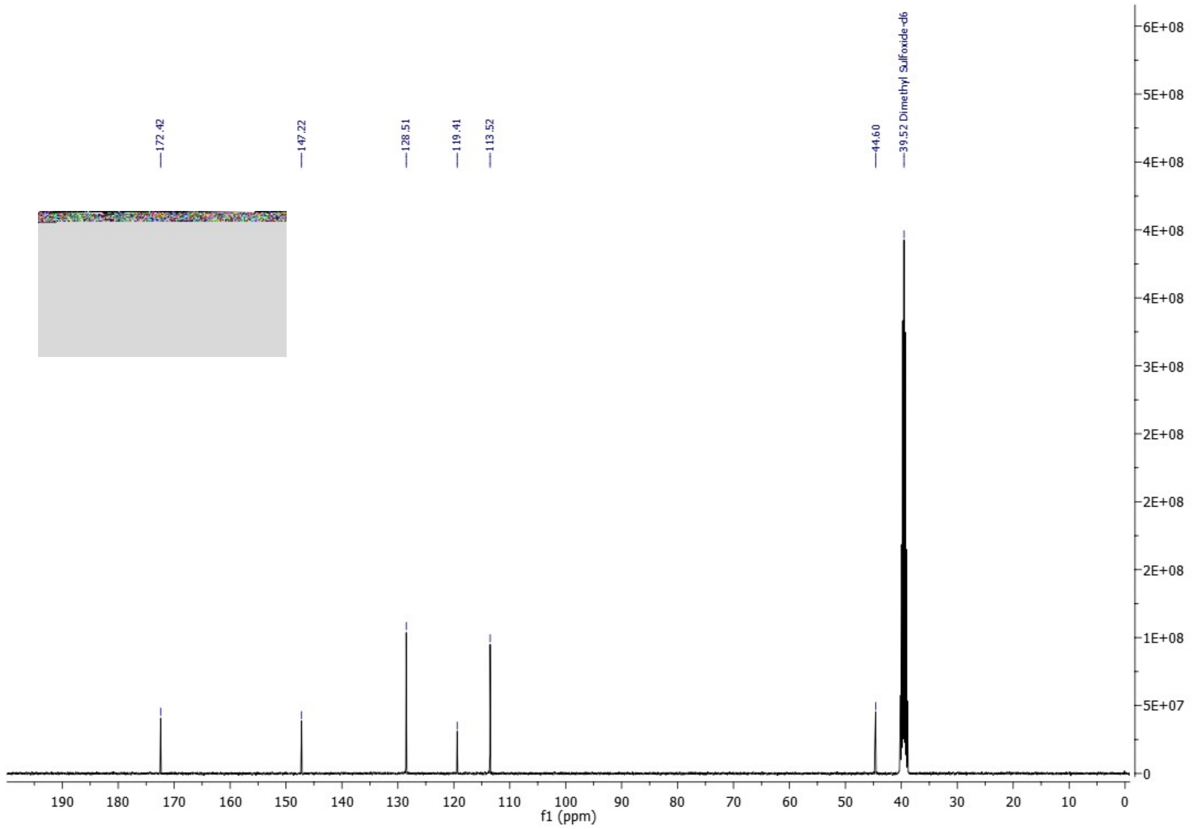

Figure S2: ^{13}C NMR of *N*-Phenylglycine (3a)

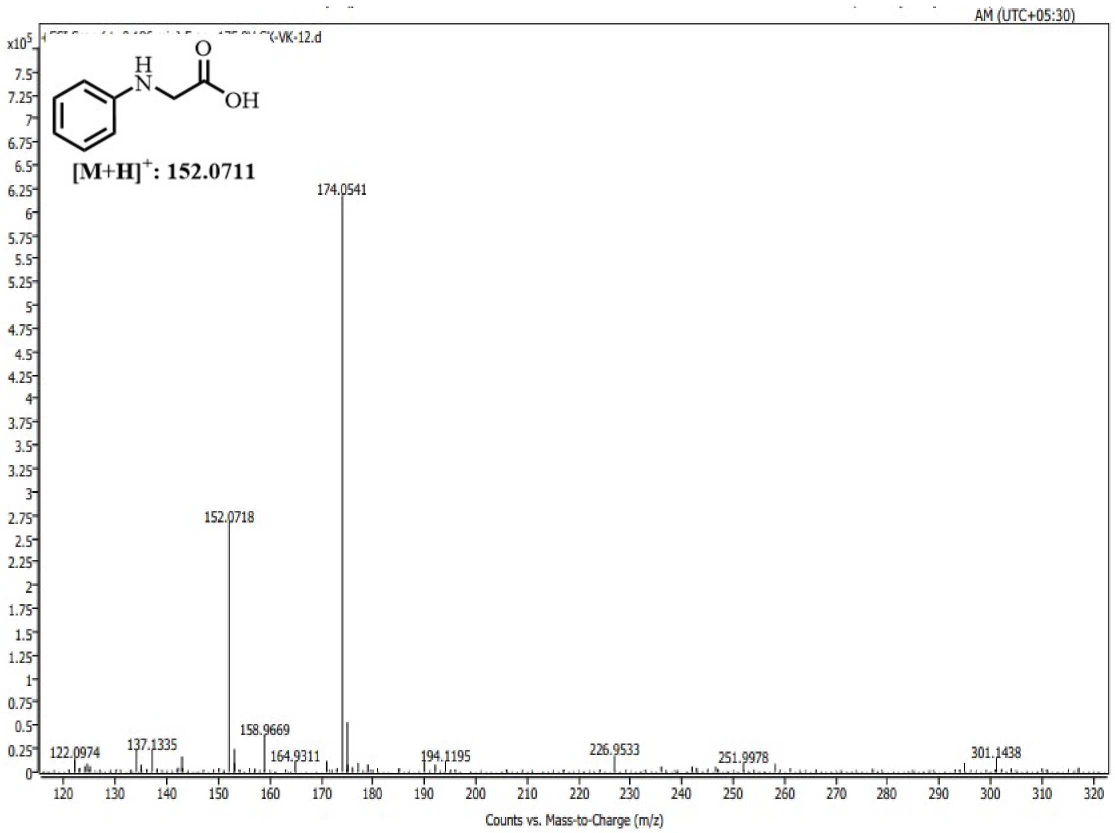

Figure S3: HRMS of *N*-Phenylglycine (3a)

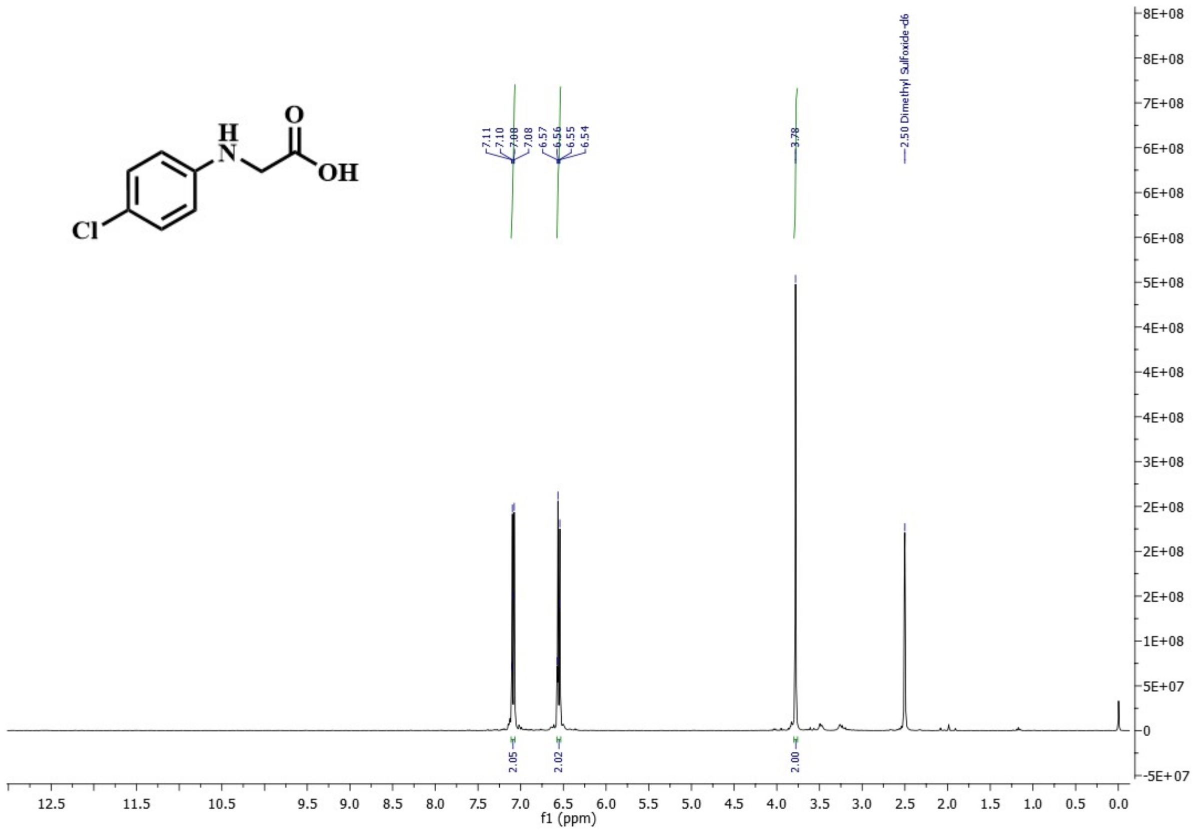

Figure S4: ^{1}H NMR of *N*-(4-Chlorophenyl)glycine (3b)

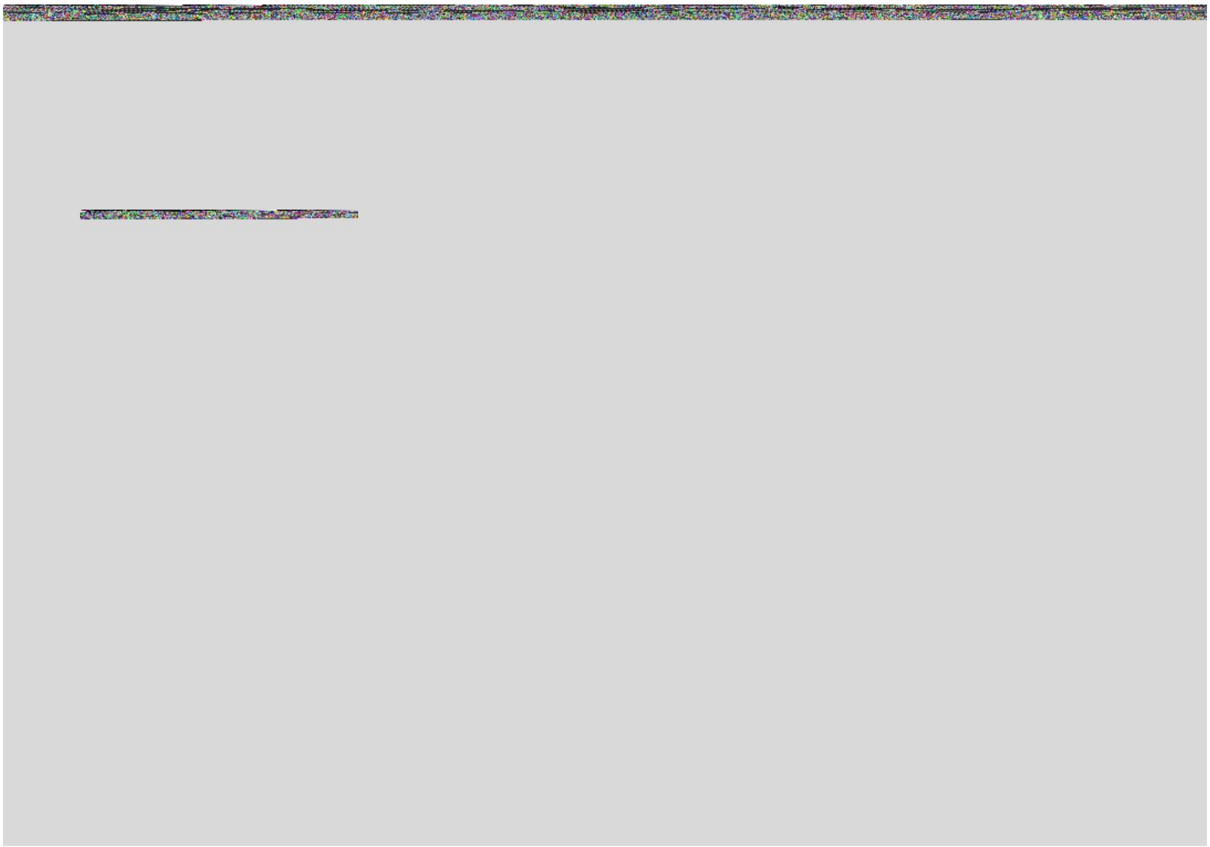

Figure S5: ^{13}C NMR of *N*-(4-Chlorophenyl)glycine (3b)

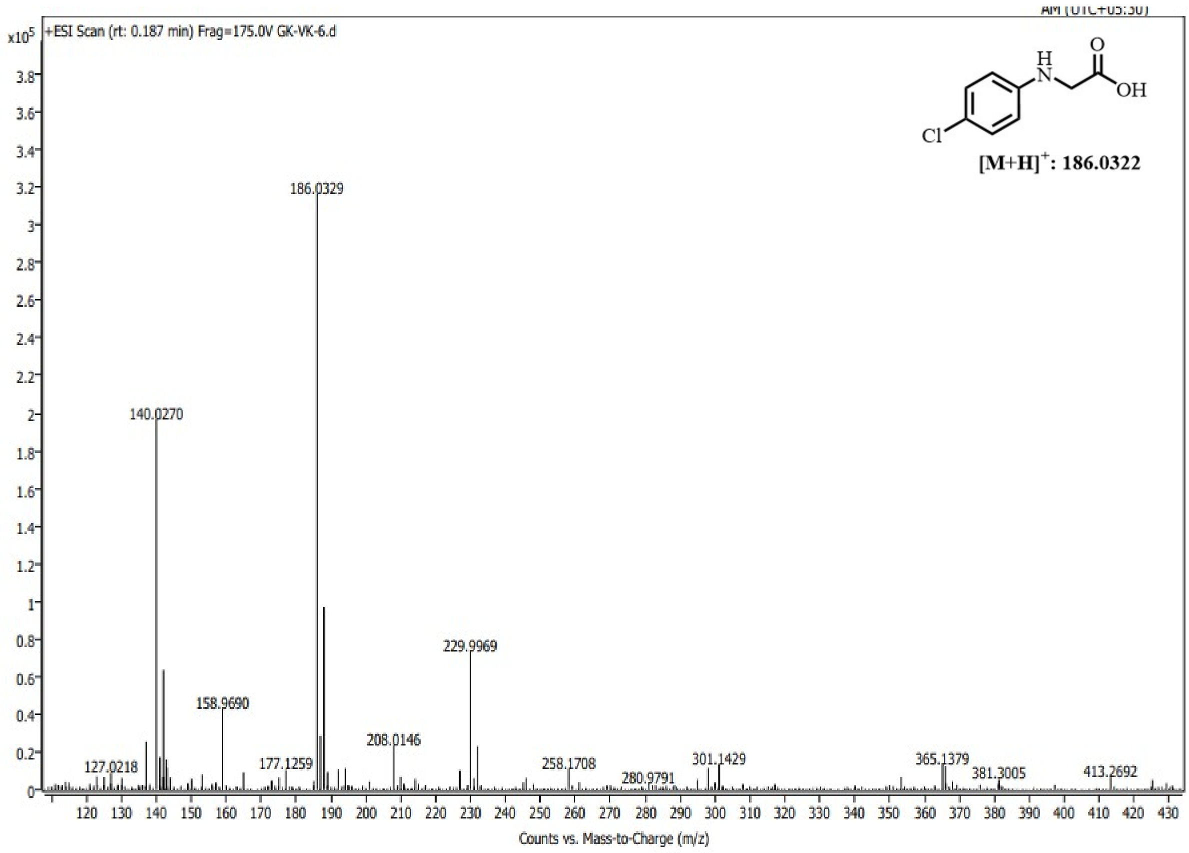

Figure S6: HRMS of *N*-(4-Chlorophenyl)glycine (3b)

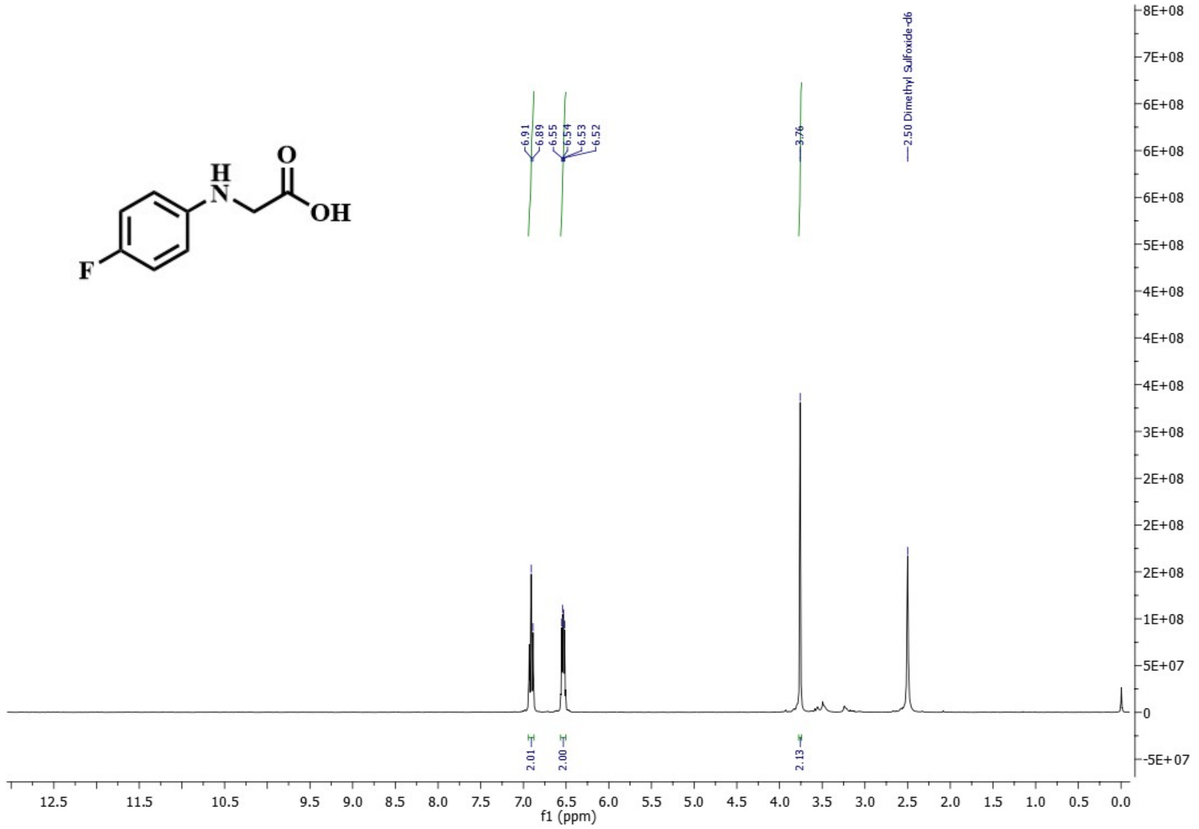

Figure S7: ^{1}H NMR of *N*-(4-Fluorophenyl)glycine (3c)

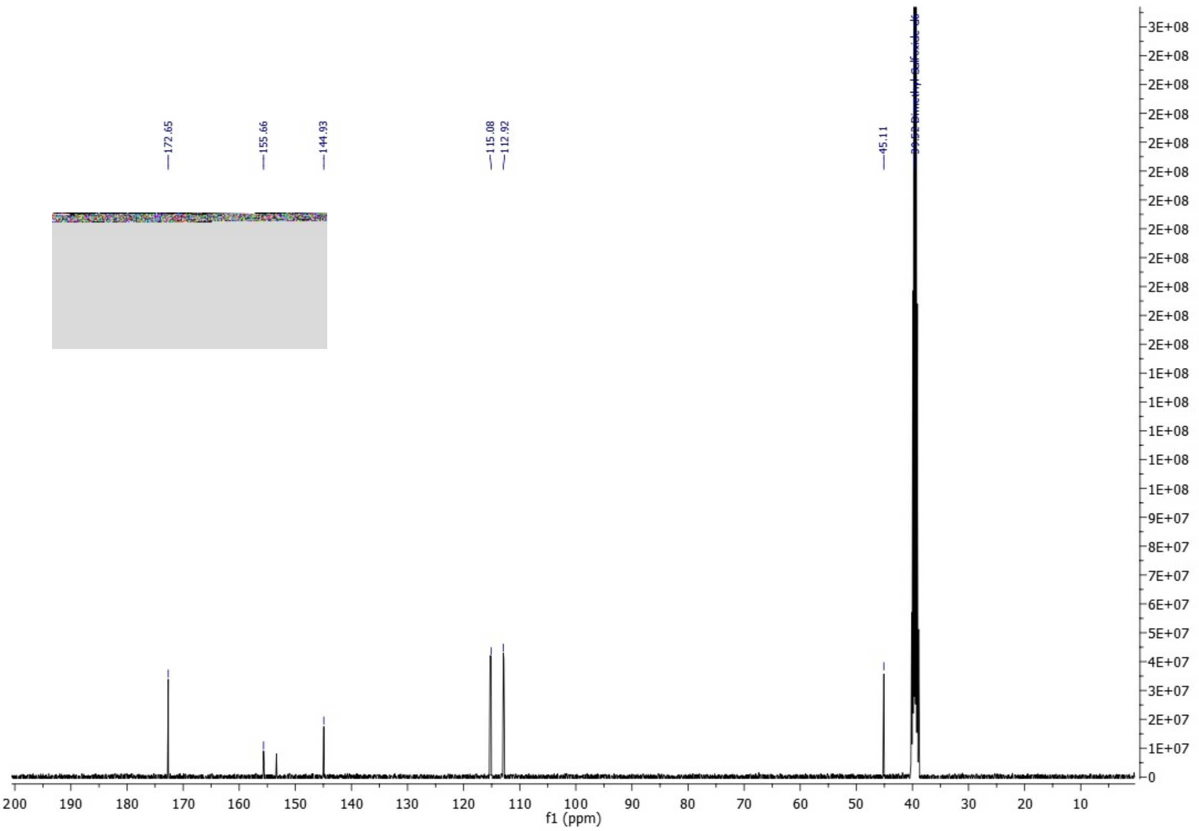

Figure S8: ^{13}C NMR of *N*-(4-Fluorophenyl)glycine (3c)

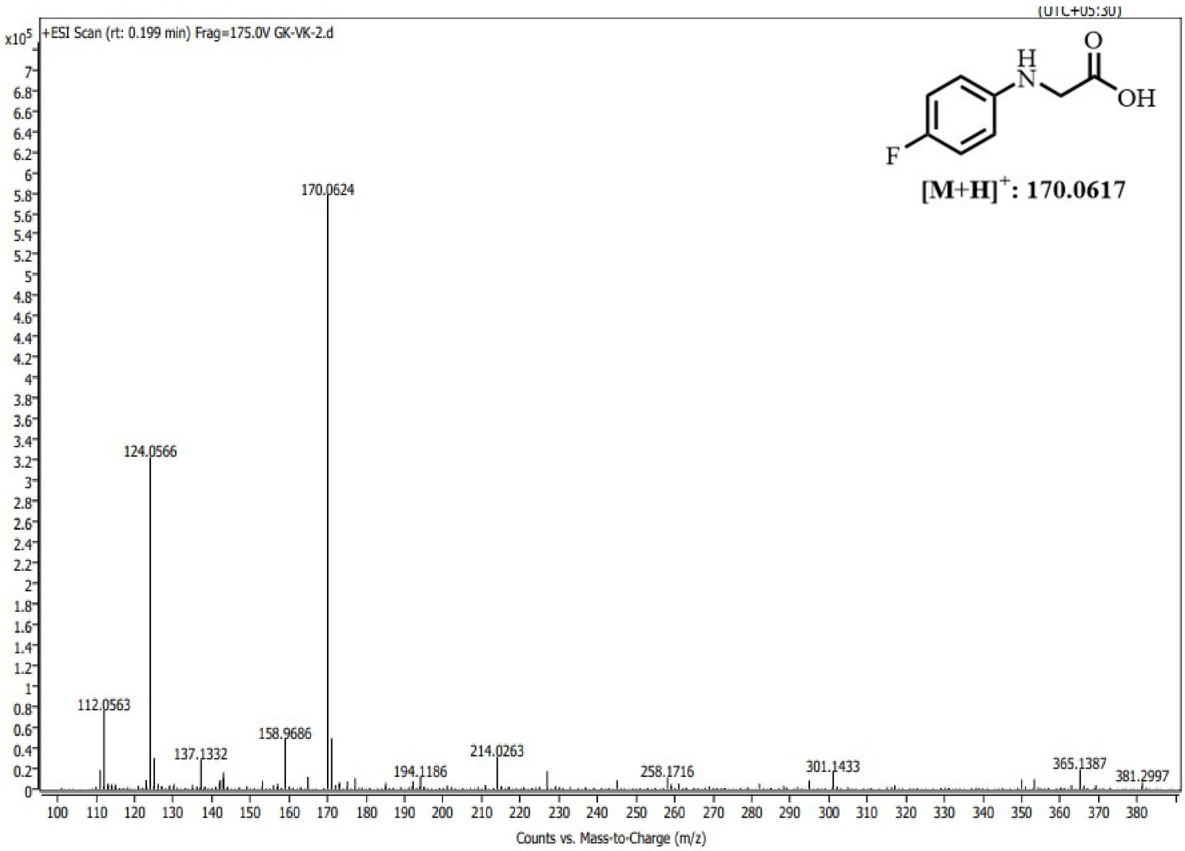

Figure S9: HRMS of *N*-(4-Fluorophenyl)glycine (3c)

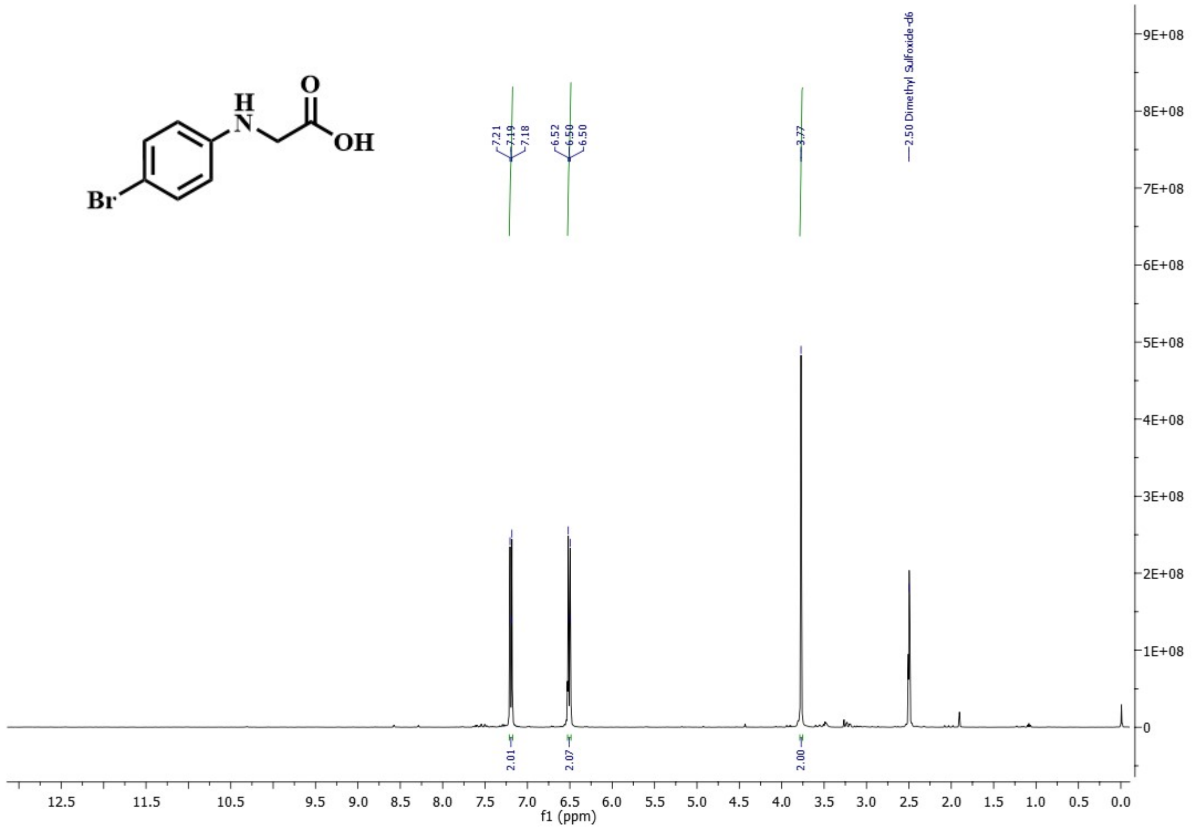

Figure S10: ^{1}H NMR of *N*-(4-Bromophenyl)glycine (3d)

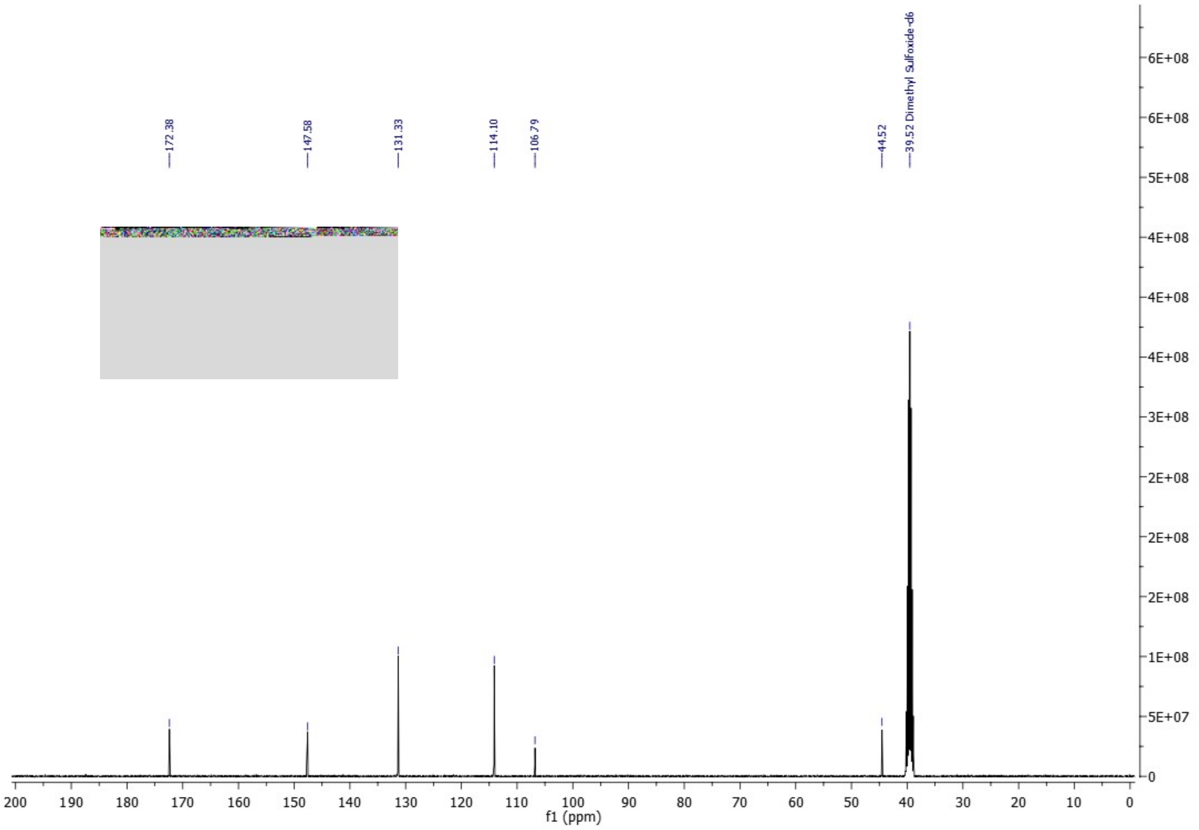

Figure S11: ^{13}C NMR of *N*-(4-Bromophenyl)glycine (3d)

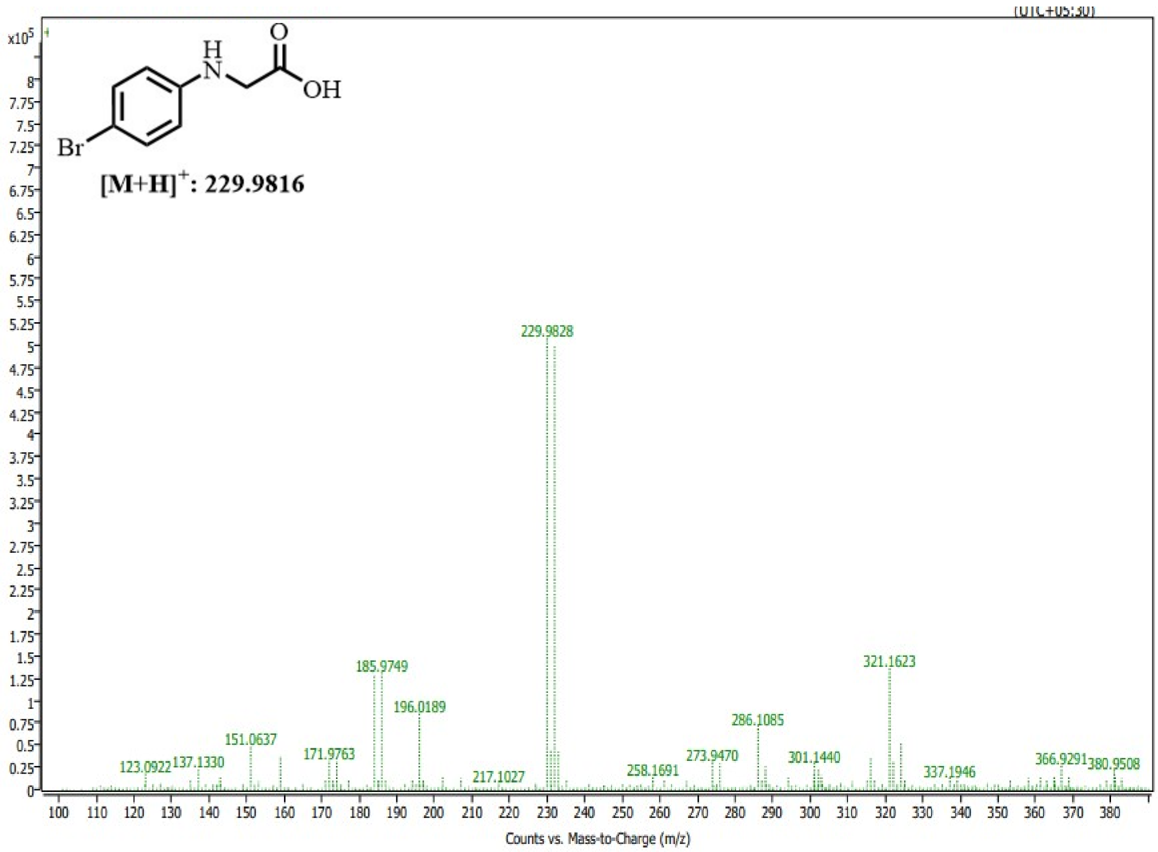

Figure S12: HRMS of *N*-(4-Bromophenyl)glycine (3d)

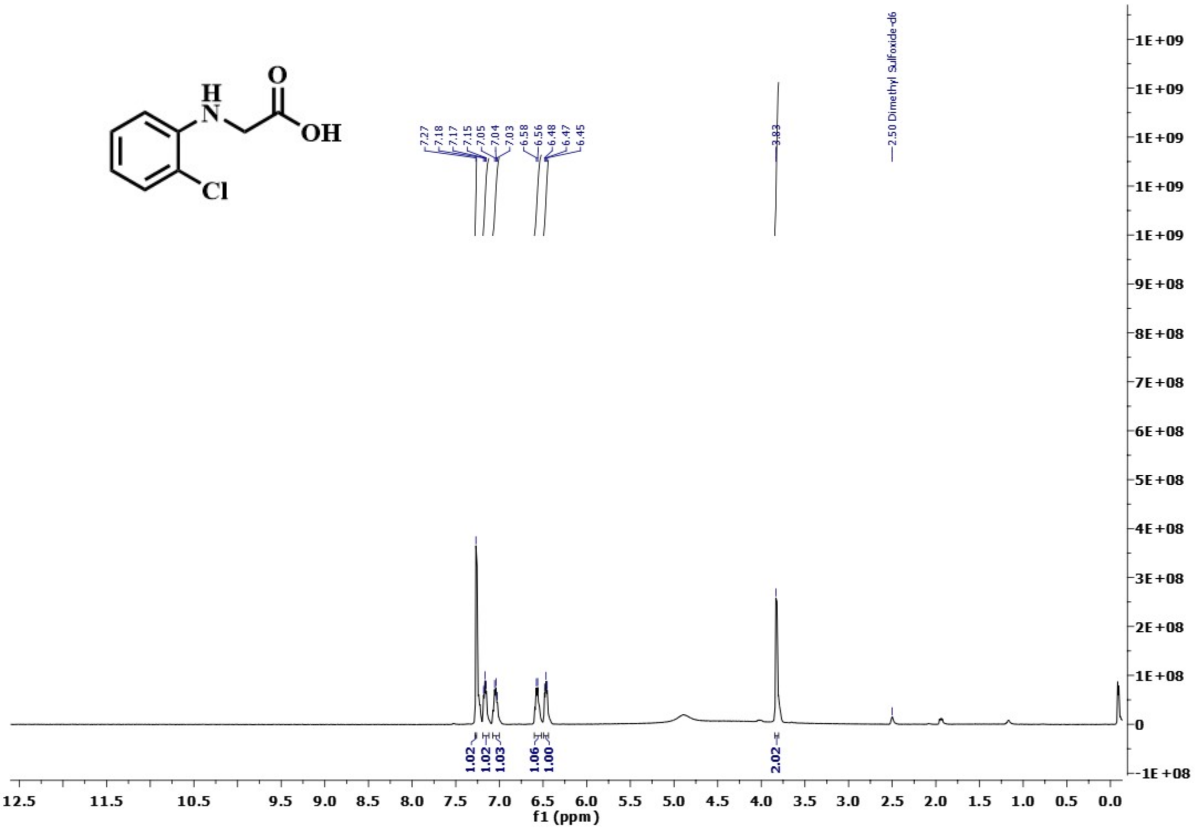

Figure S13: ^{1}H NMR of *N*-(2-Chlorophenyl)glycine (3e)

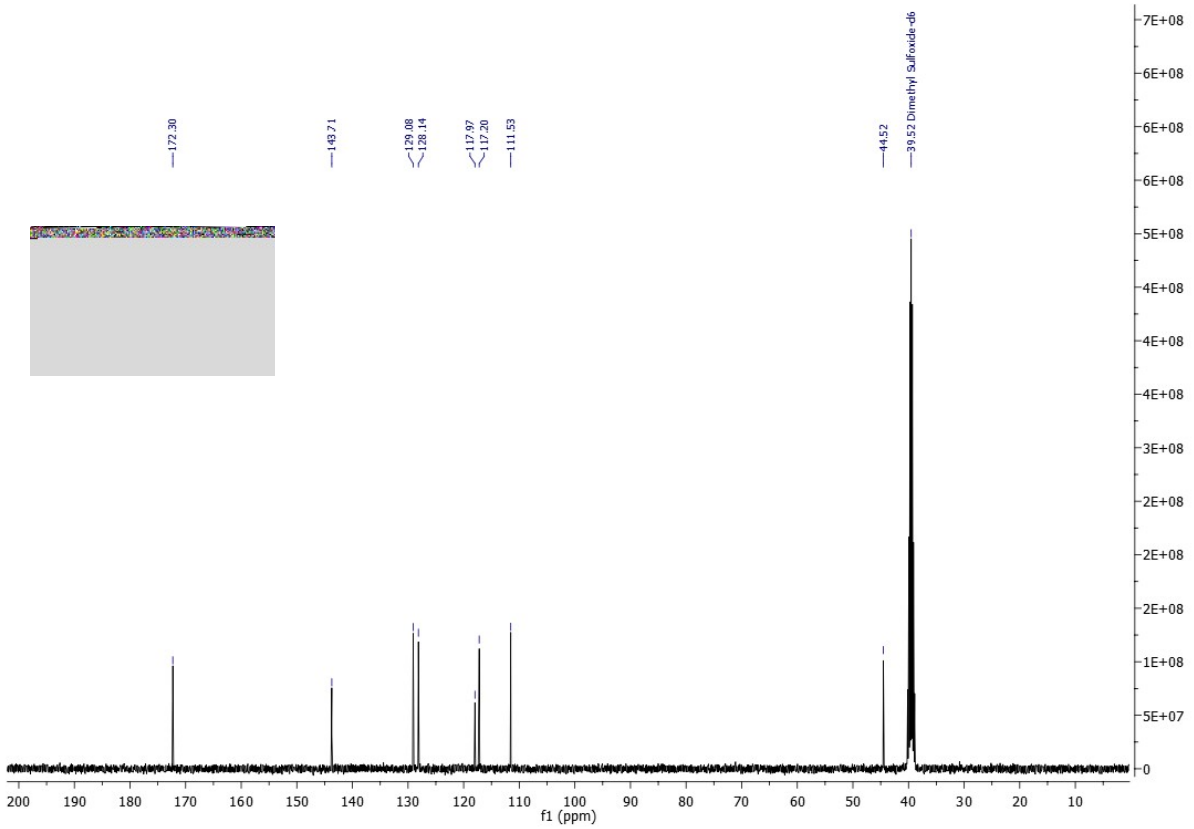

Figure S14: ^{13}C NMR of *N*-(2-Chlorophenyl)glycine (3e)

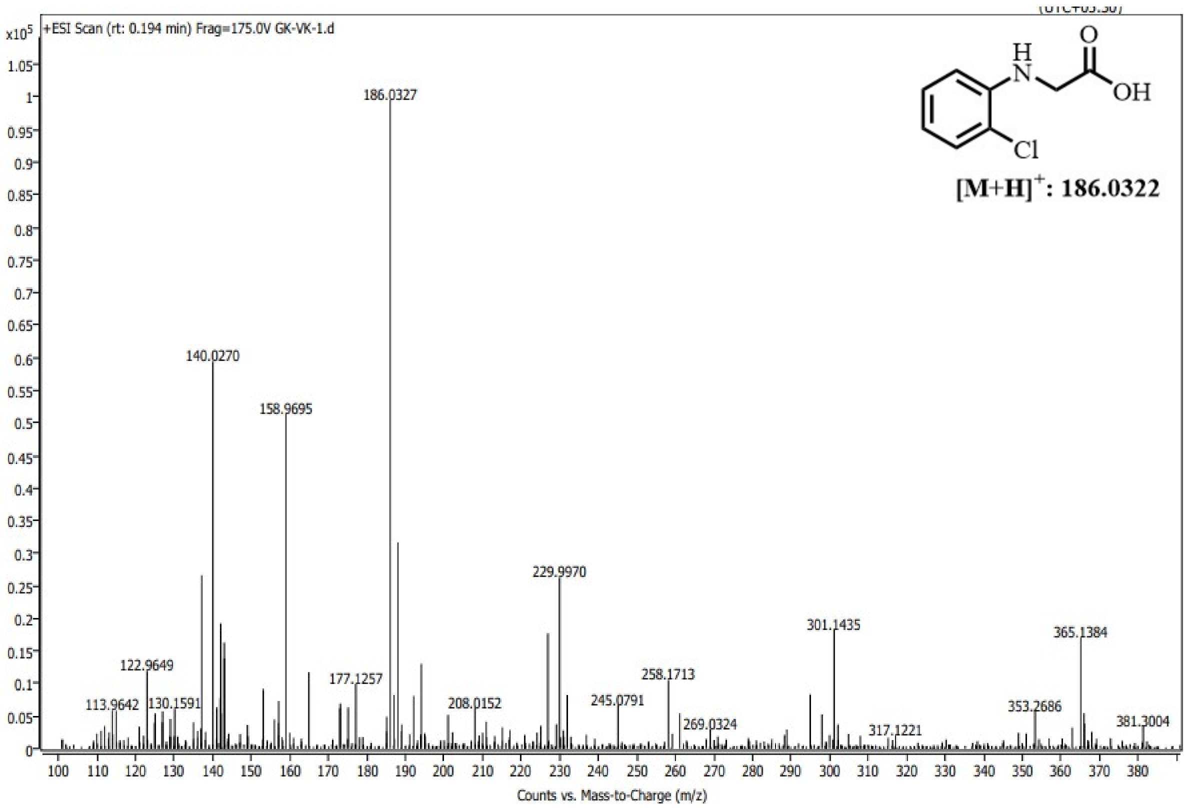

Figure S15: HRMS of *N*-(2-Chlorophenyl)glycine (3e)

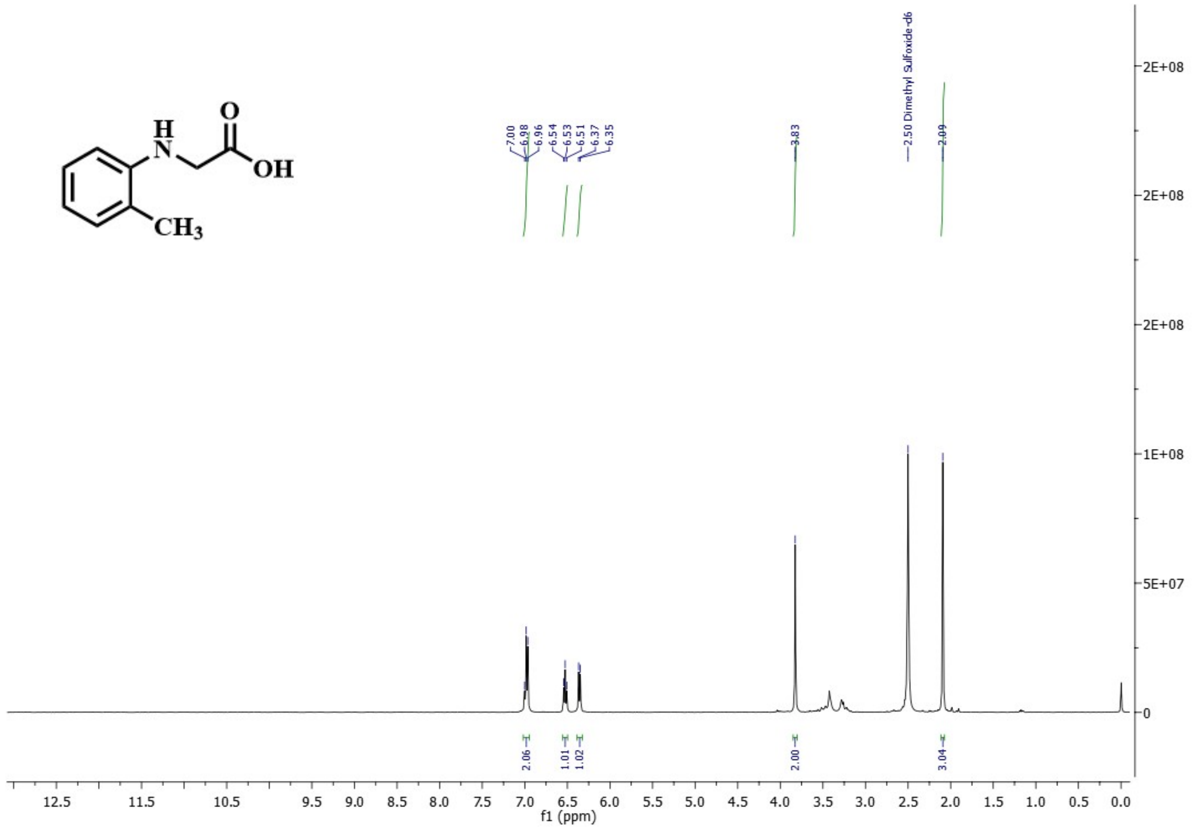

Figure S16: ^{1}H NMR of *N*-(*o*-Tolyl)glycine (3f)

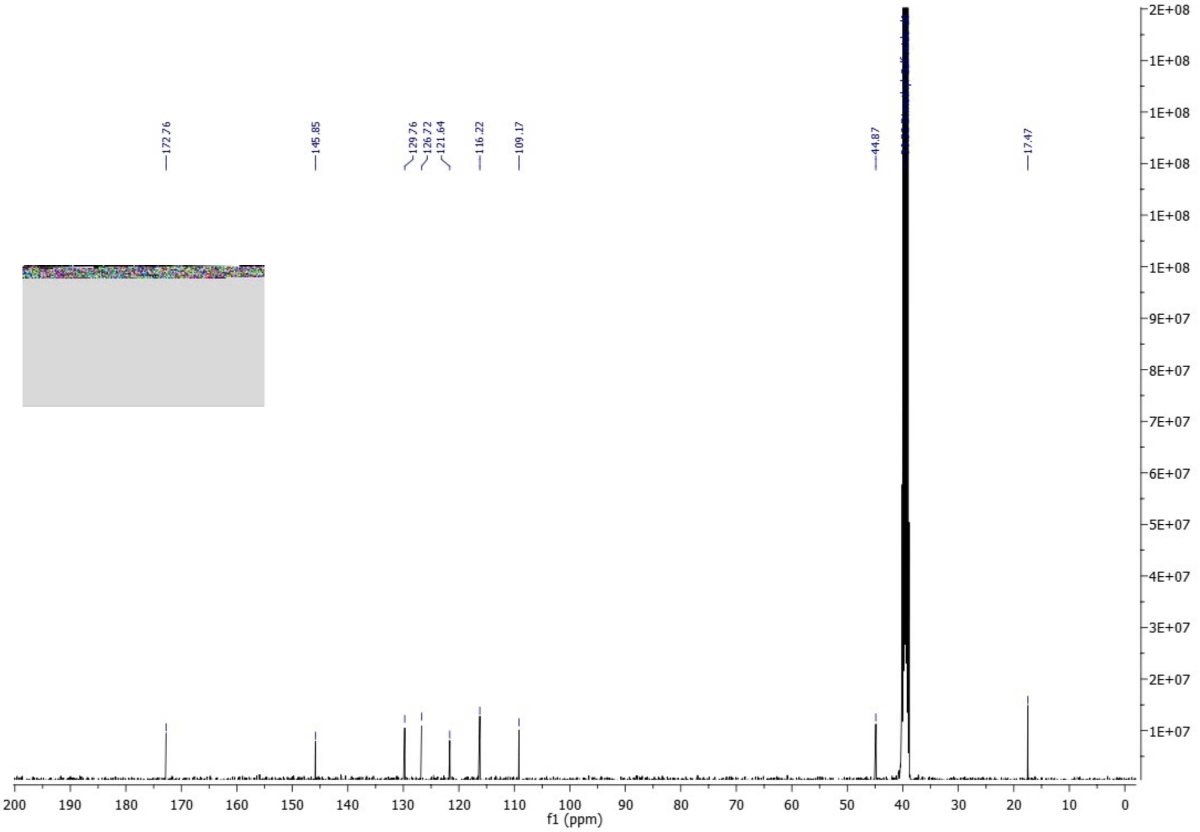

Figure S17: ^{13}C NMR of *N*-(*o*-Tolyl)glycine (3f)

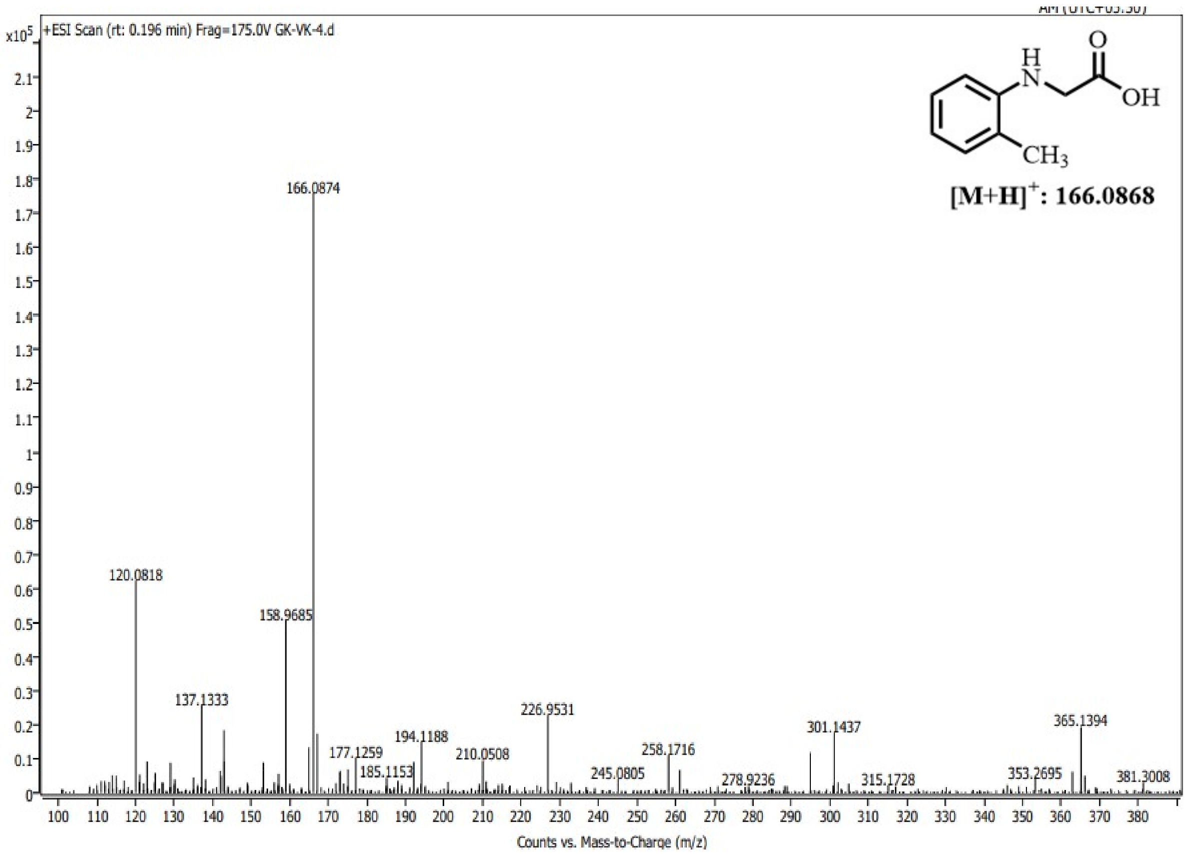

Figure S18: HRMS of *N*-(*o*-Tolyl)glycine (3f)

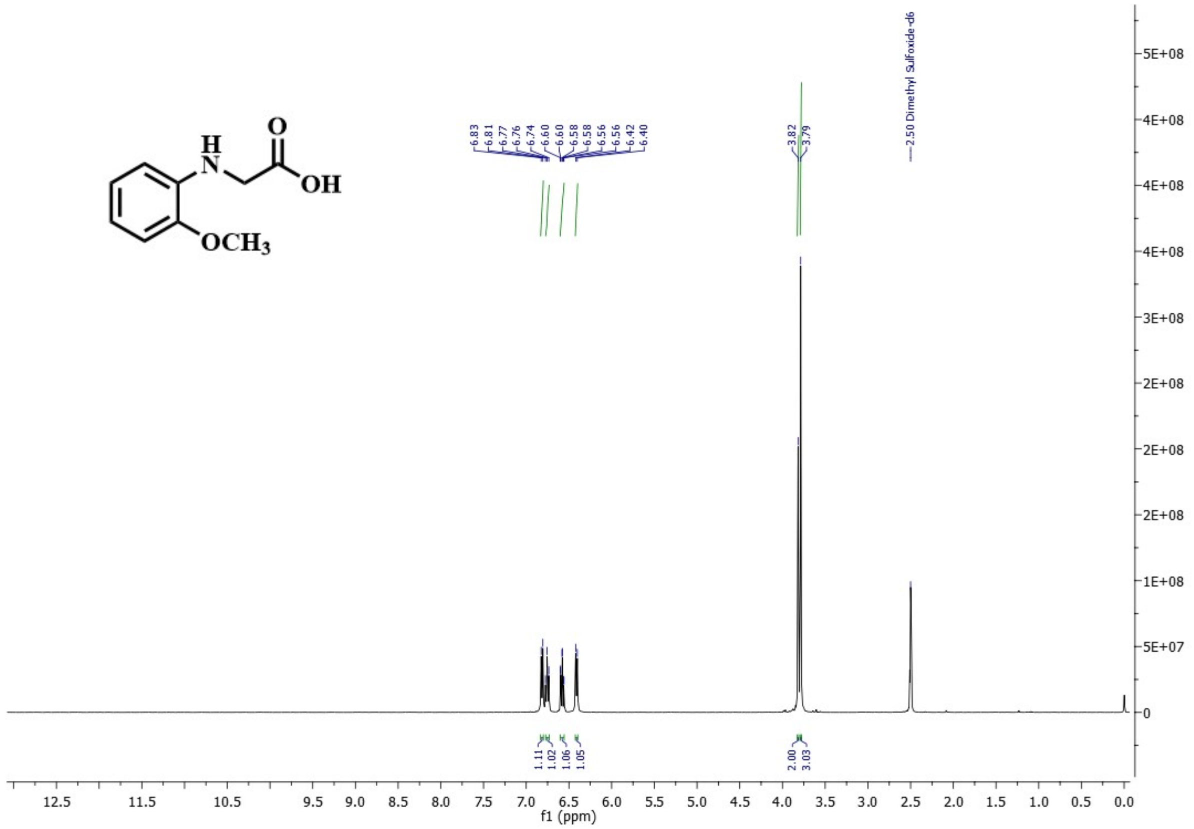

Figure S19: ^{1}H NMR of *N*-(2-Methoxyphenyl)glycine (3g)

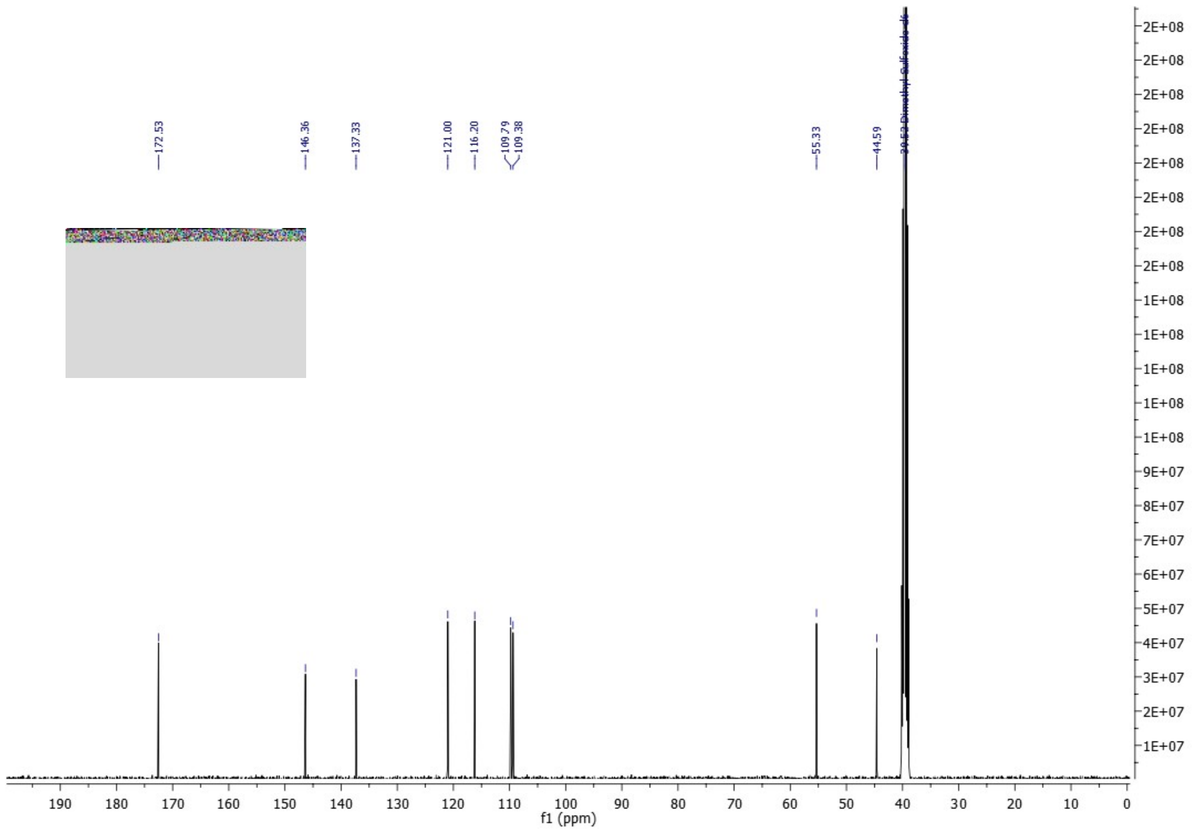

Figure S20: ^{13}C NMR of *N*-(2-Methoxyphenyl)glycine (3g)

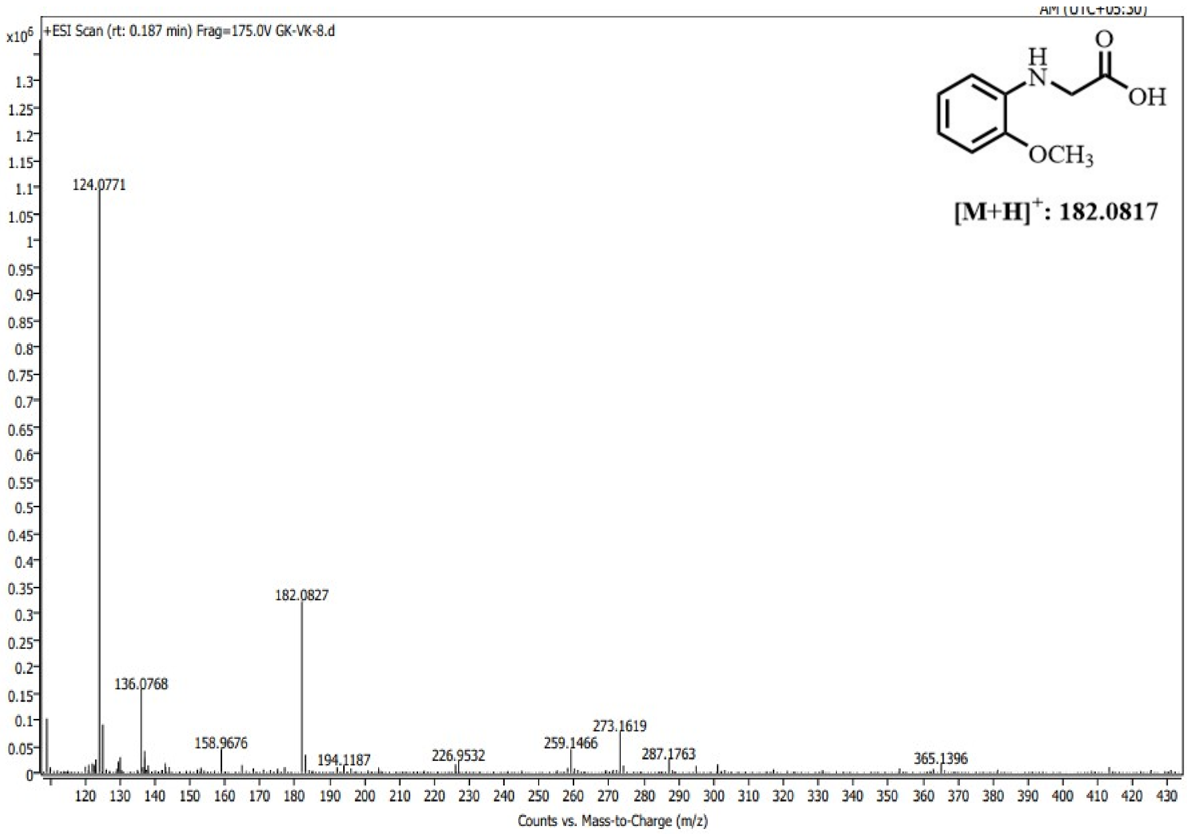

Figure S21: HRMS of *N*-(2-Methoxyphenyl)glycine (3g)

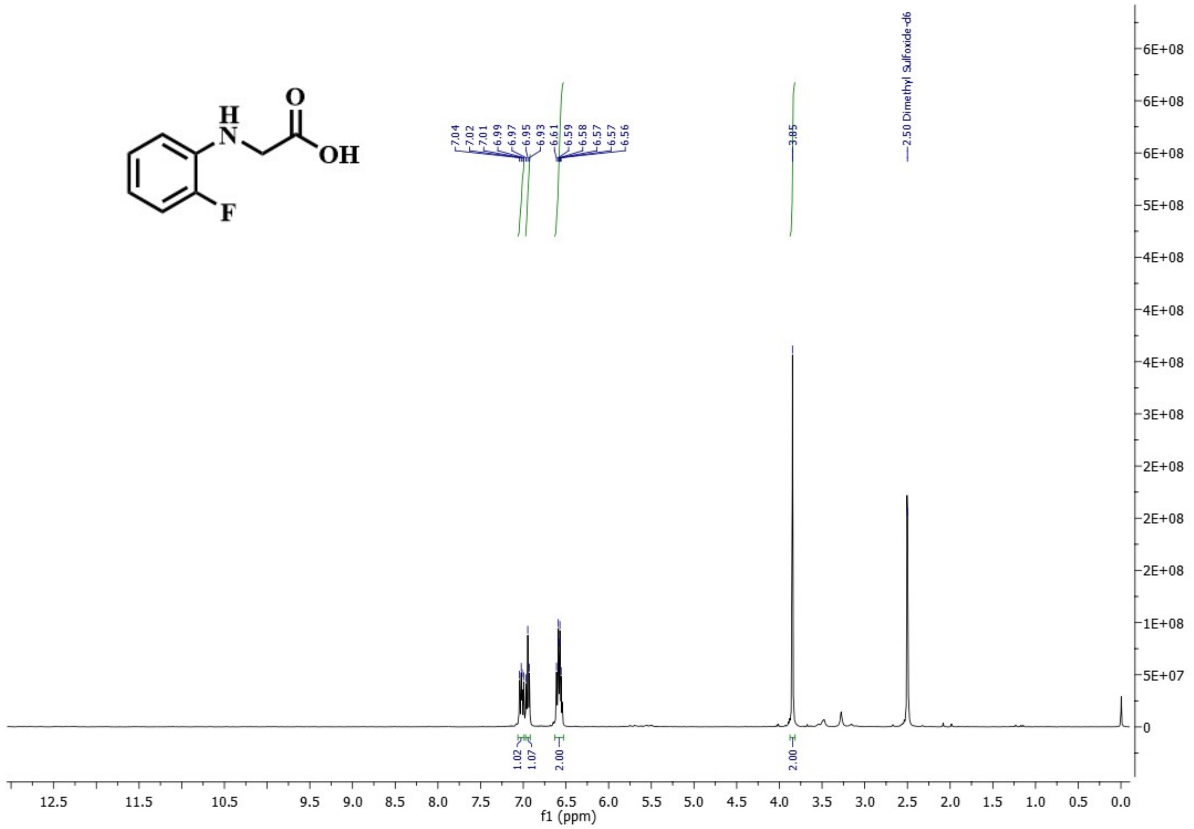

Figure S22: ^{1}H NMR of *N*-(2-Fluorophenyl)glycine (3h)

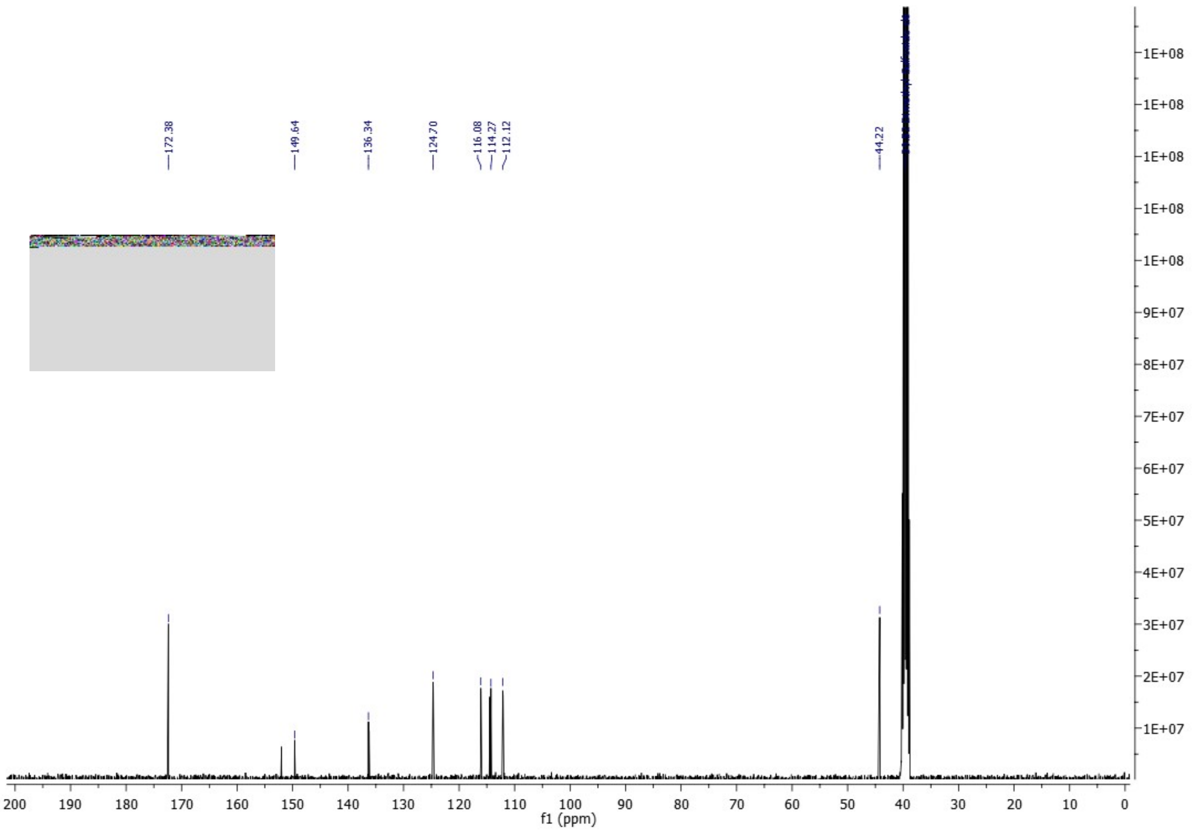

Figure S23: ^{13}C NMR of *N*-(2-Fluorophenyl)glycine (3h)

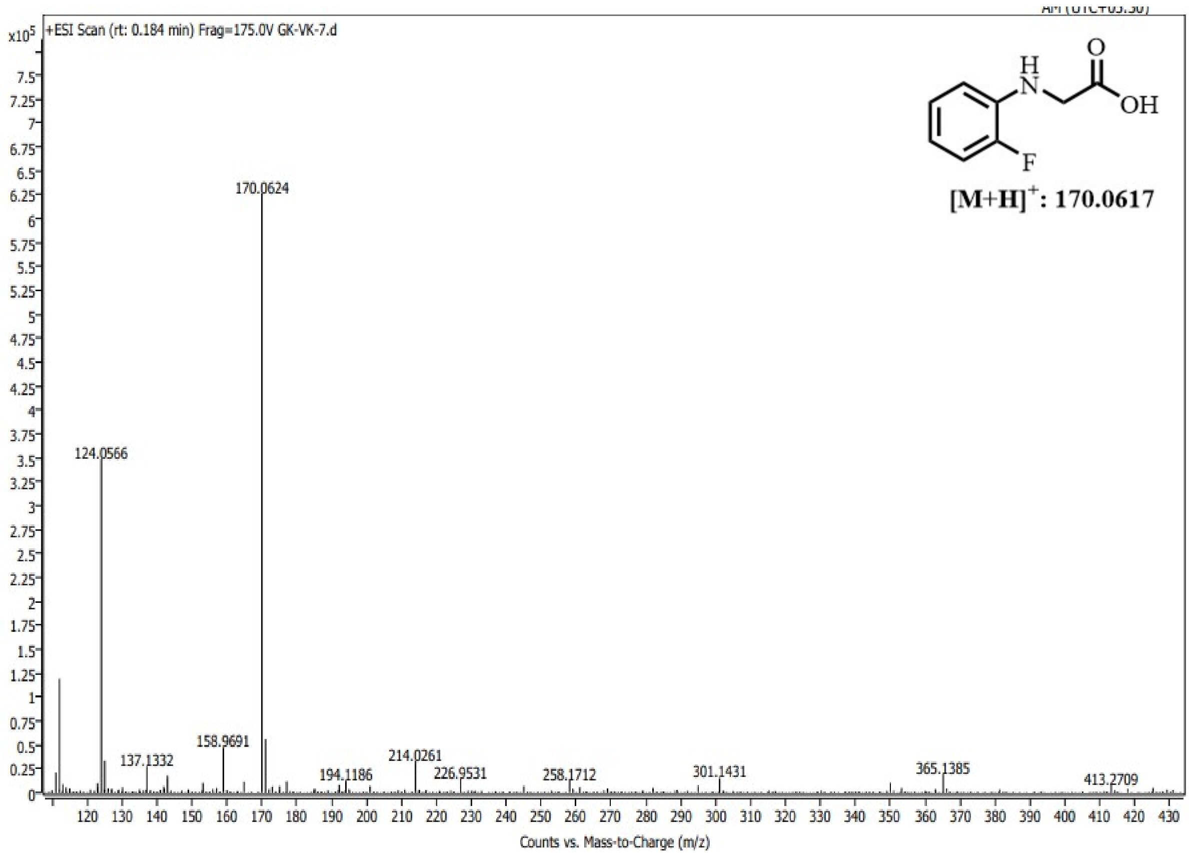

Figure S24: HRMS of *N*-(2-Fluorophenyl)glycine (3h)

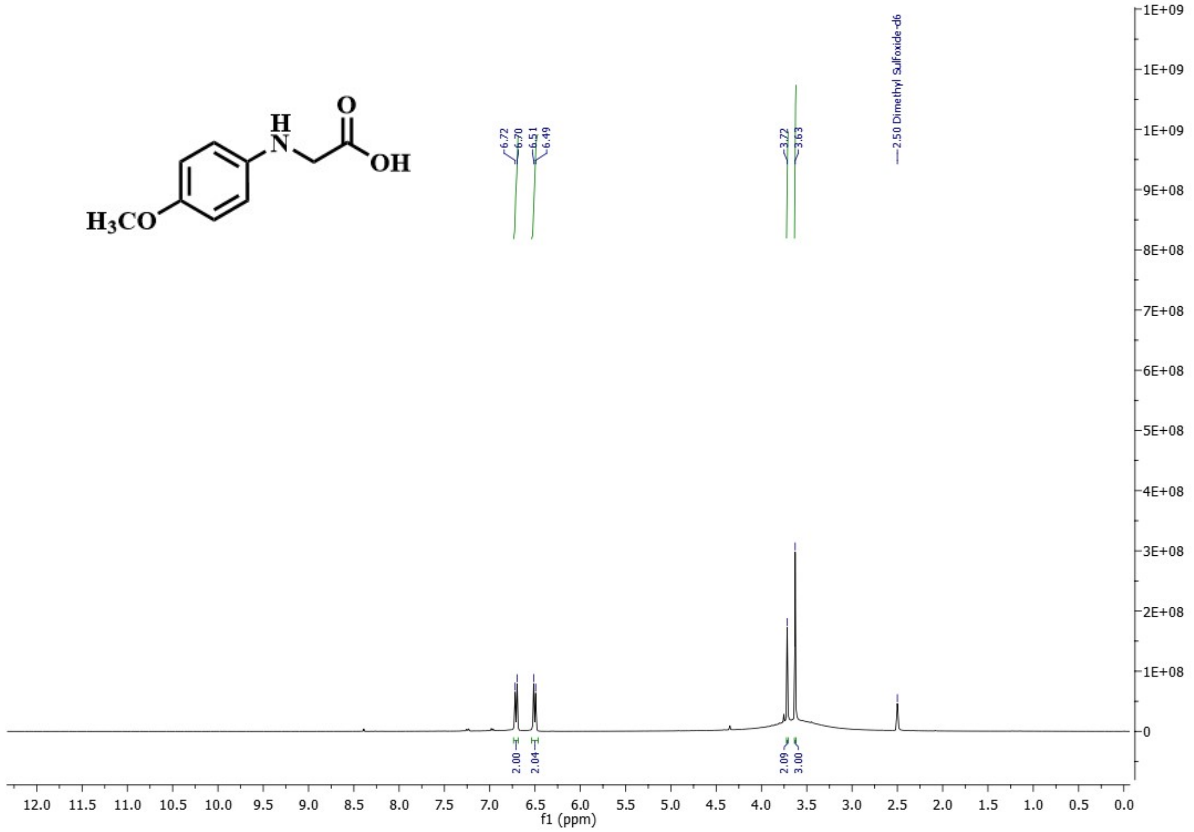

Figure S25: ^{1}H NMR of *N*-(4-Methoxyphenyl)glycine (3i)

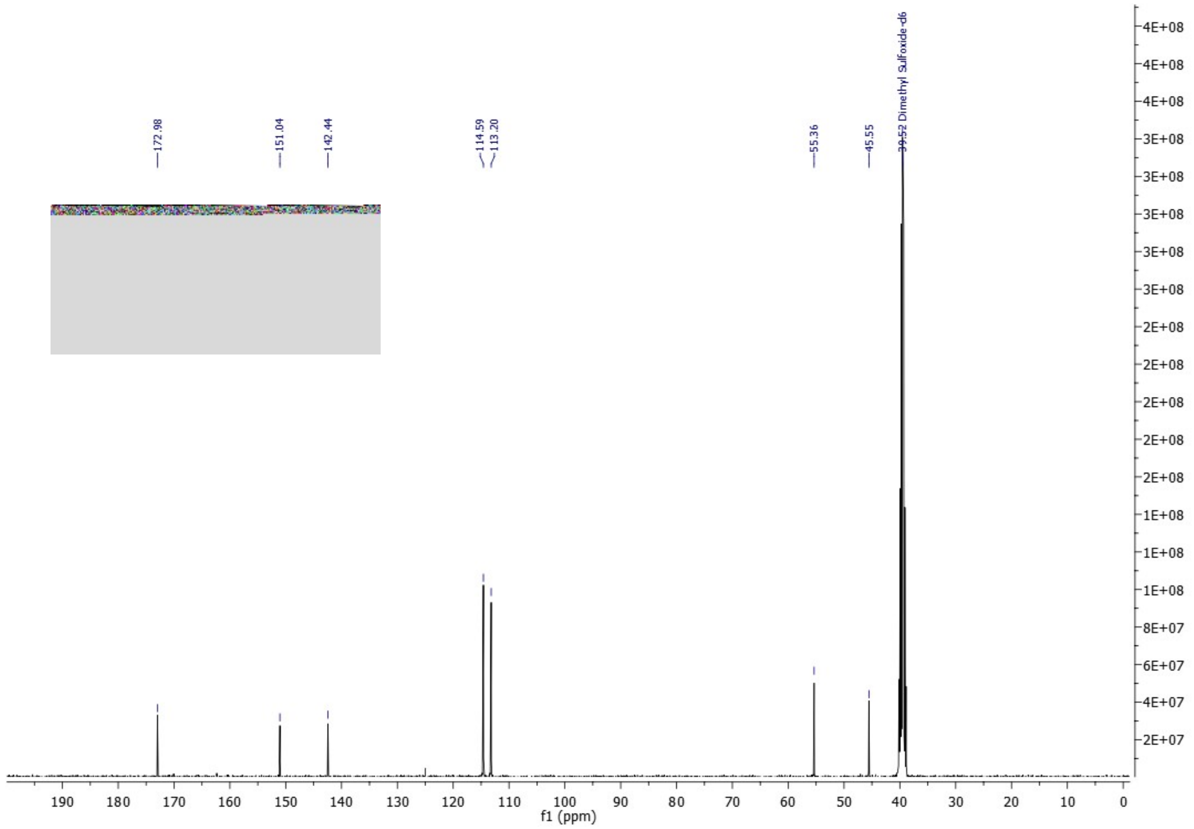

Figure S36: ^{13}C NMR of *N*-(4-Methoxyphenyl)glycine (3i)

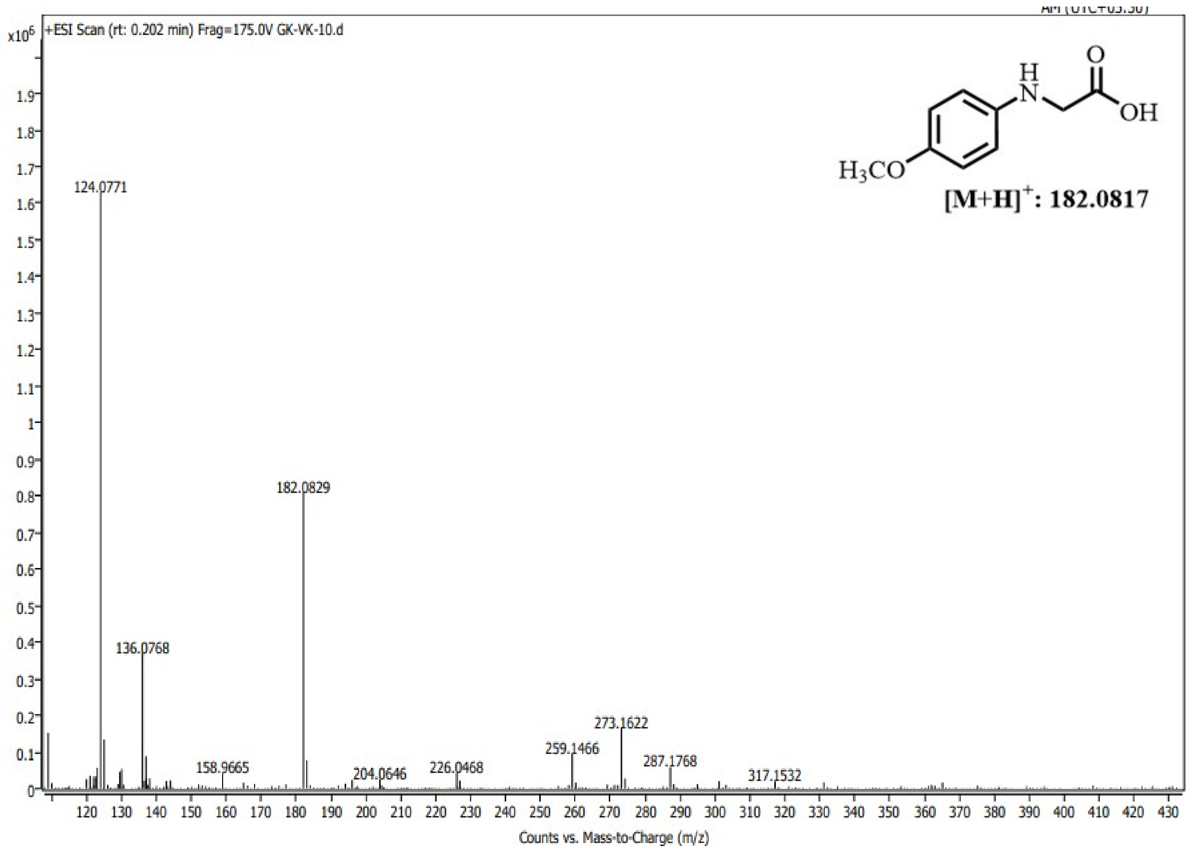

Figure S27: HRMS of *N*-(4-Methoxyphenyl)glycine (3i)

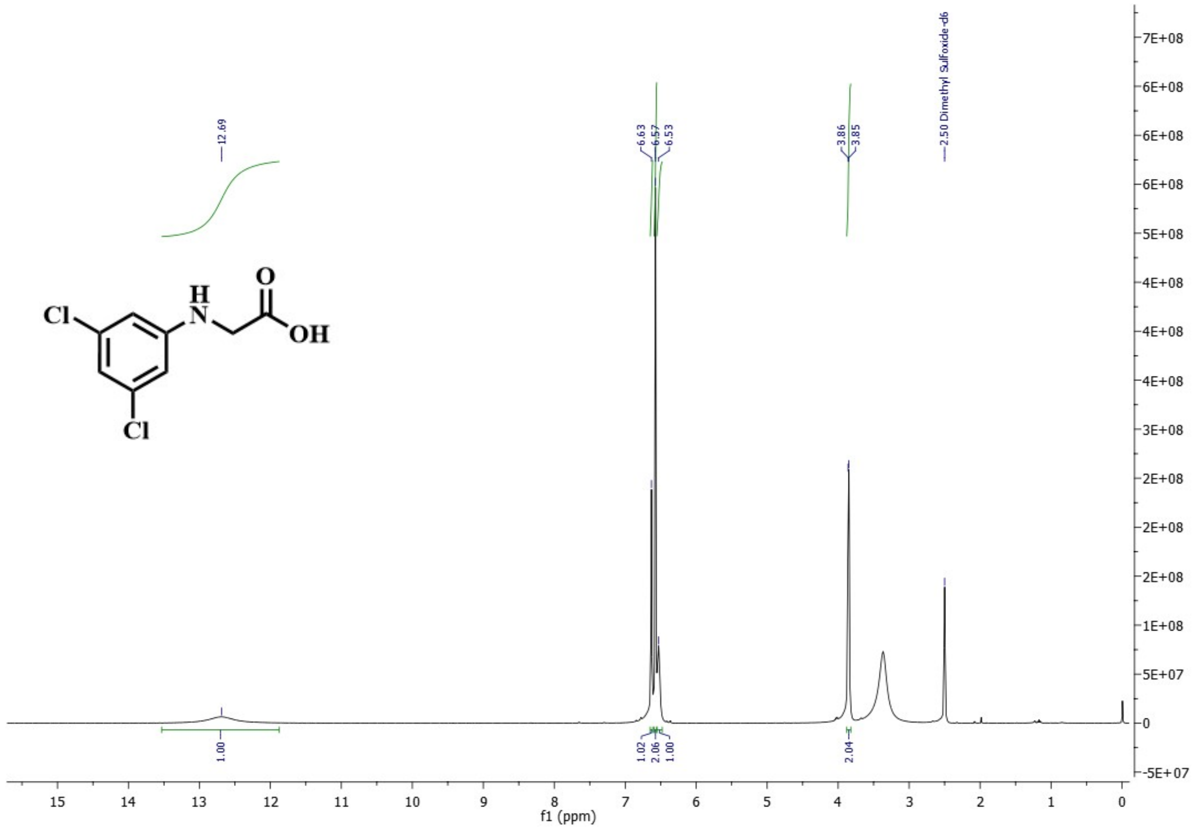

Figure S28: ^{1}H NMR of *N*-(3,5-Dichlorophenyl)glycine (3j)

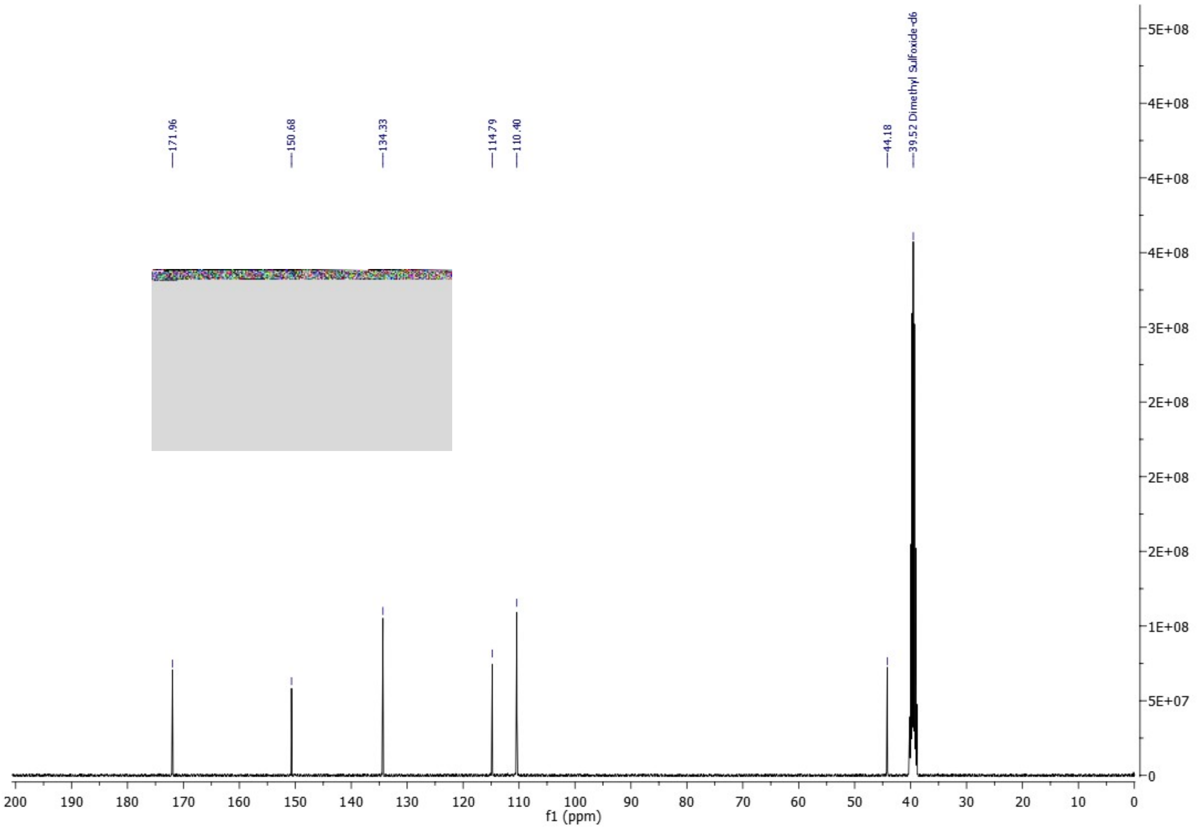

Figure S29: ^{13}C NMR of *N*-(3,5-Dichlorophenyl)glycine (3j)

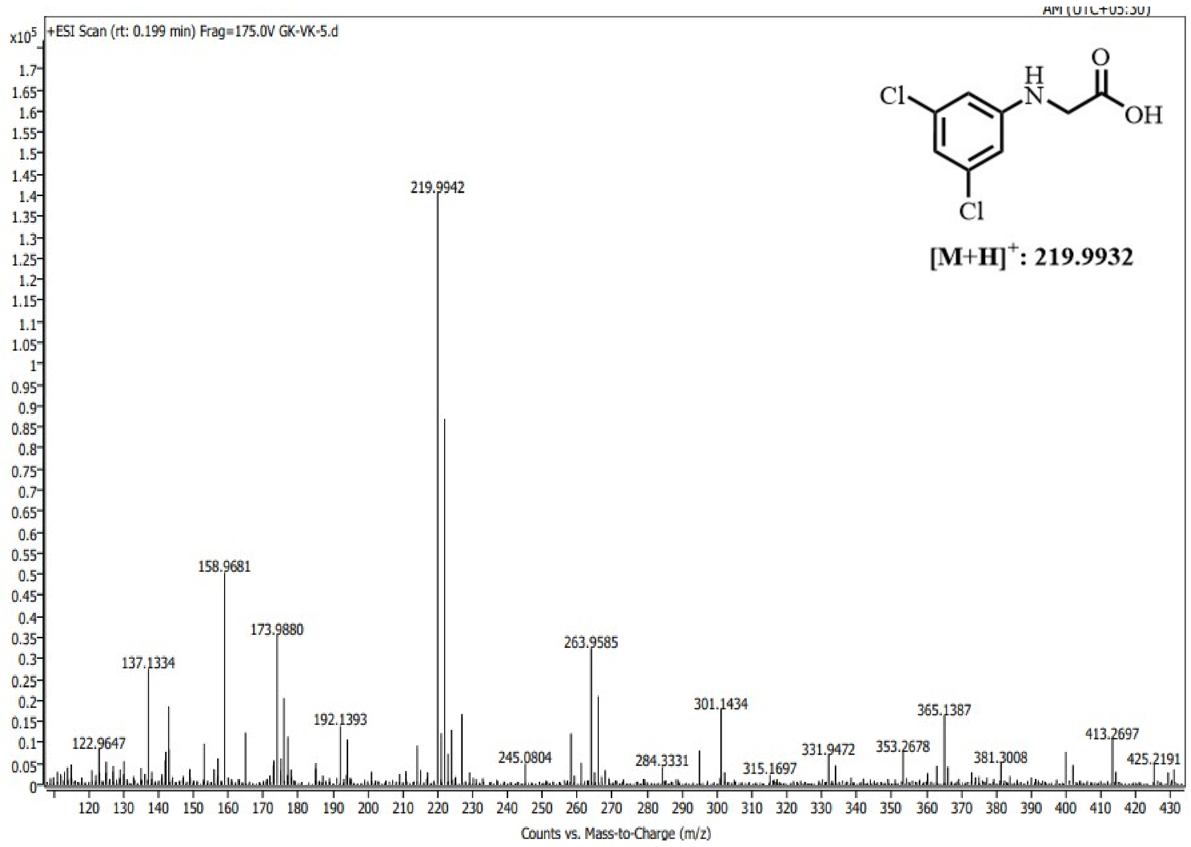

Figure S30: HRMS of *N*-(3,5-Dichlorophenyl)glycine (3j)

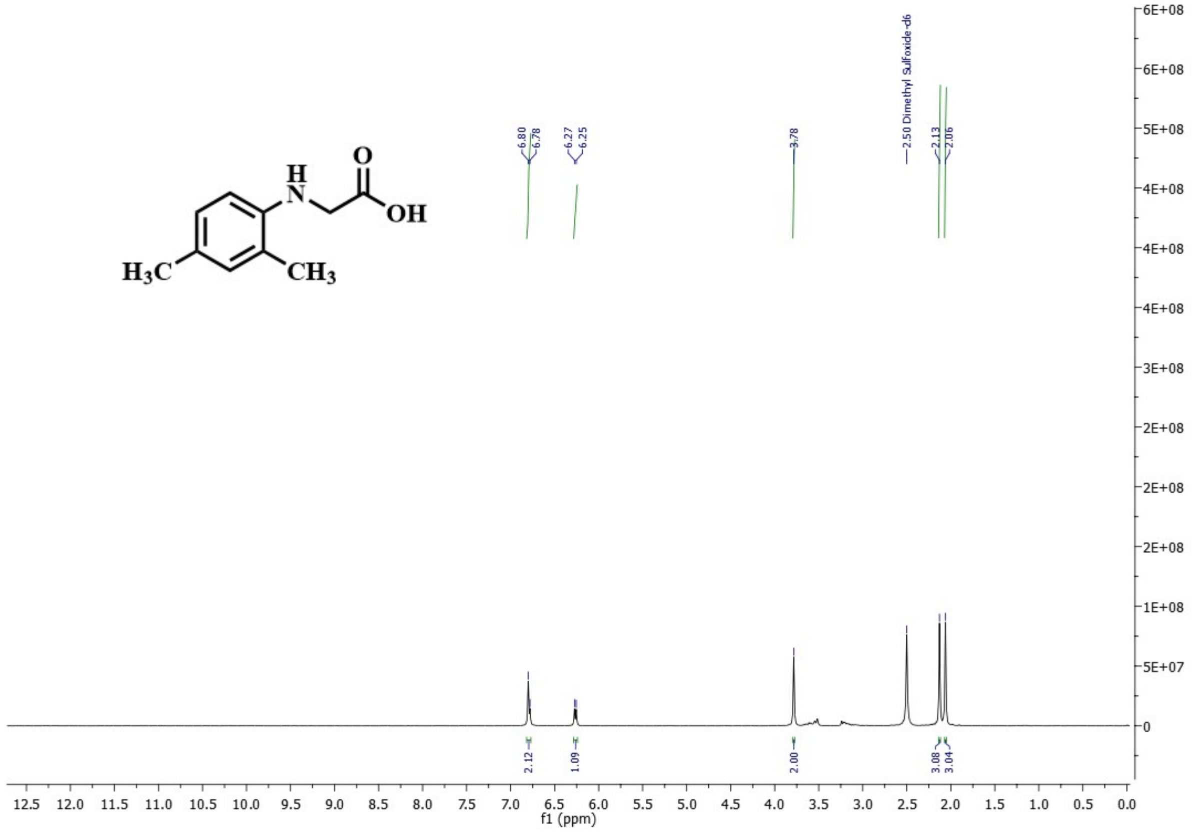

Figure S31: ^{1}H NMR of *N*-(2,4-Dimethylphenyl)glycine (3k)

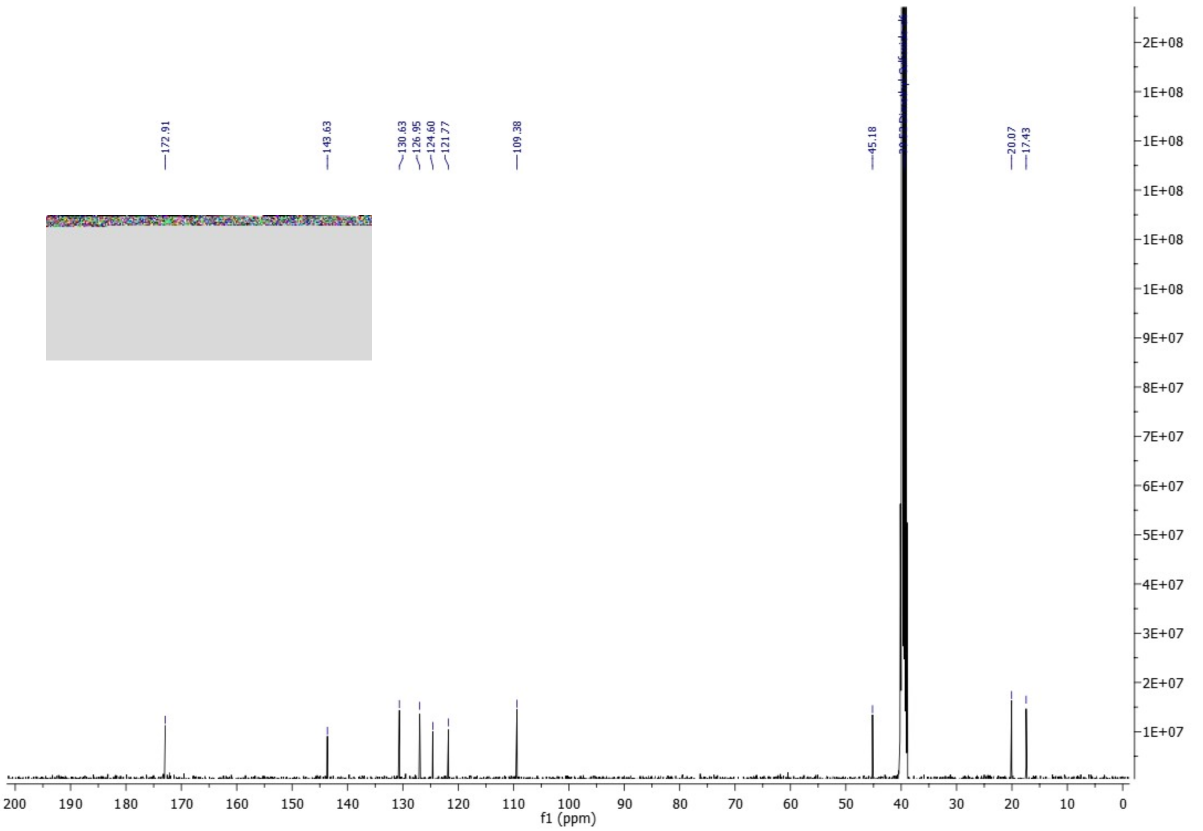

Figure S32: ^{13}C NMR of *N*-(2,4-Dimethylphenyl)glycine (3k)

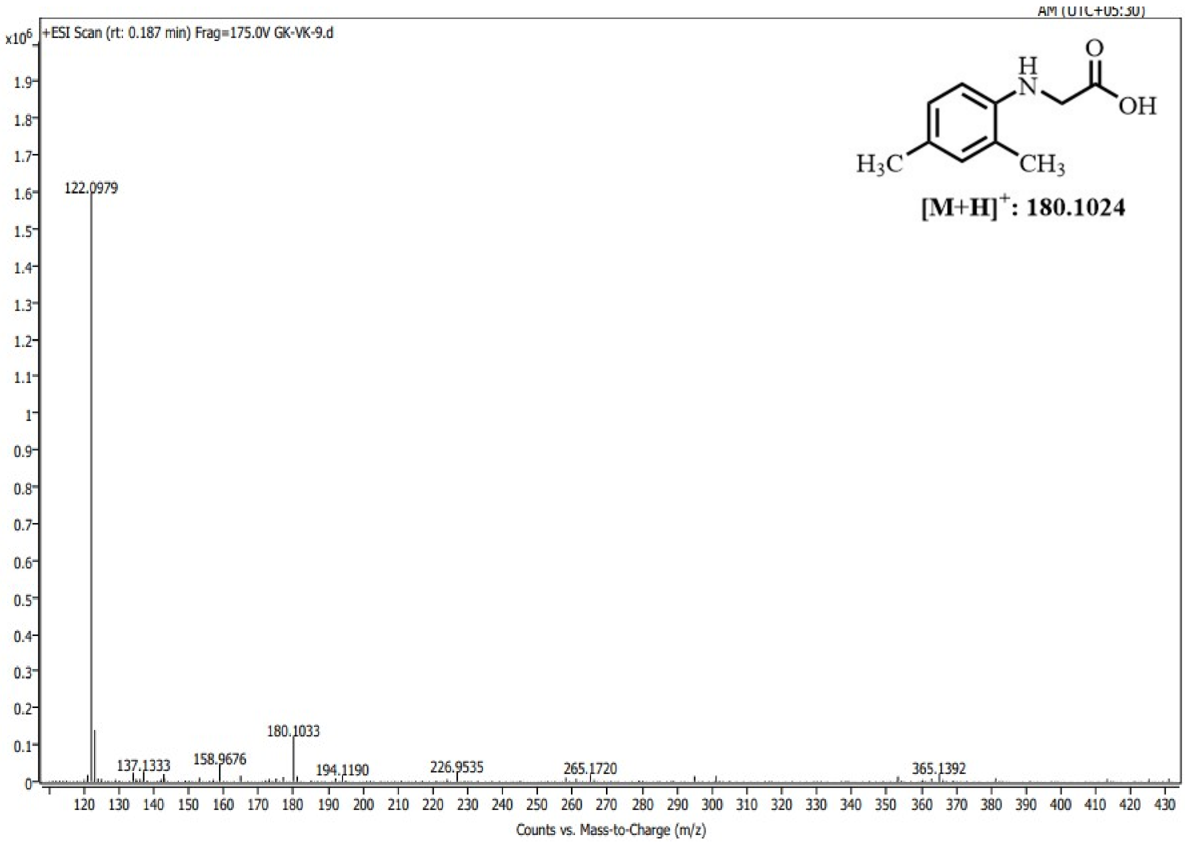

Figure S33: HRMS of *N*-(2,4-Dimethylphenyl)glycine (3k)

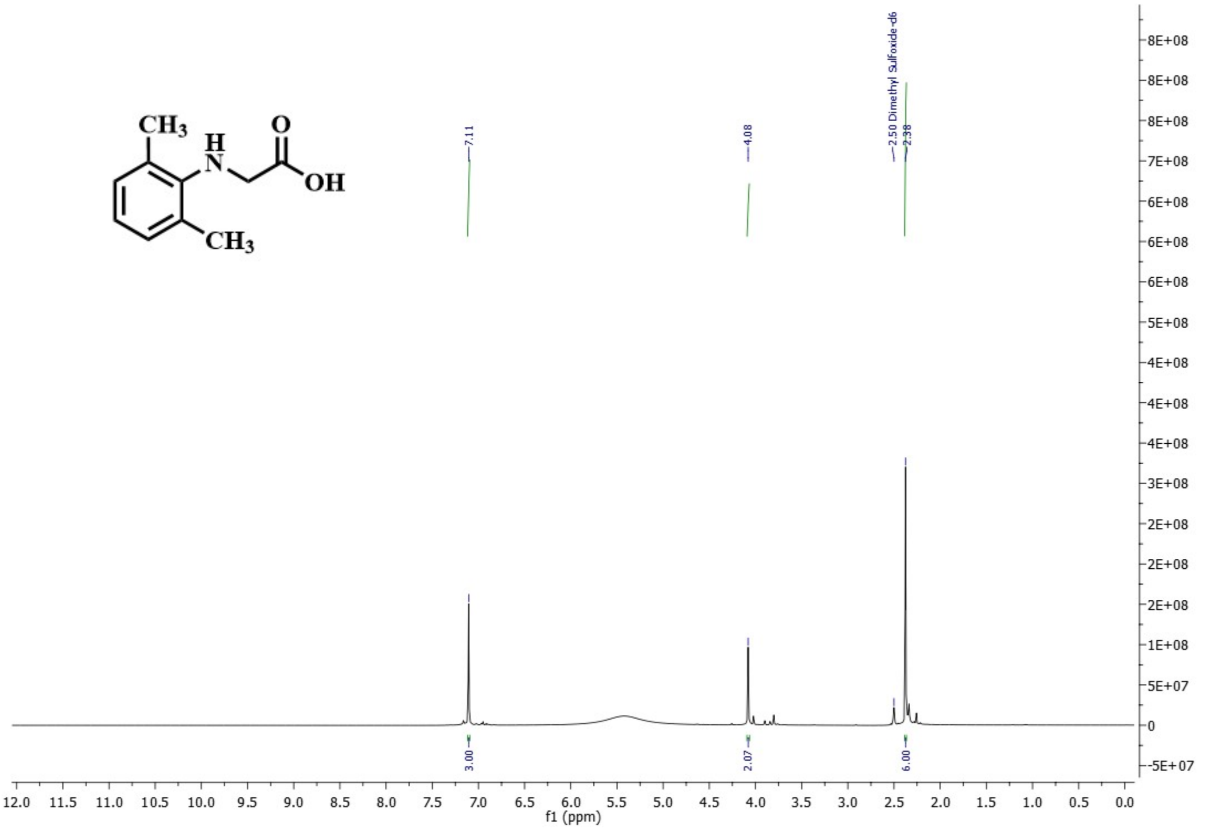

Figure S34: ^{1}H NMR of *N*-(2,6-Dimethylphenyl)glycine (3l)

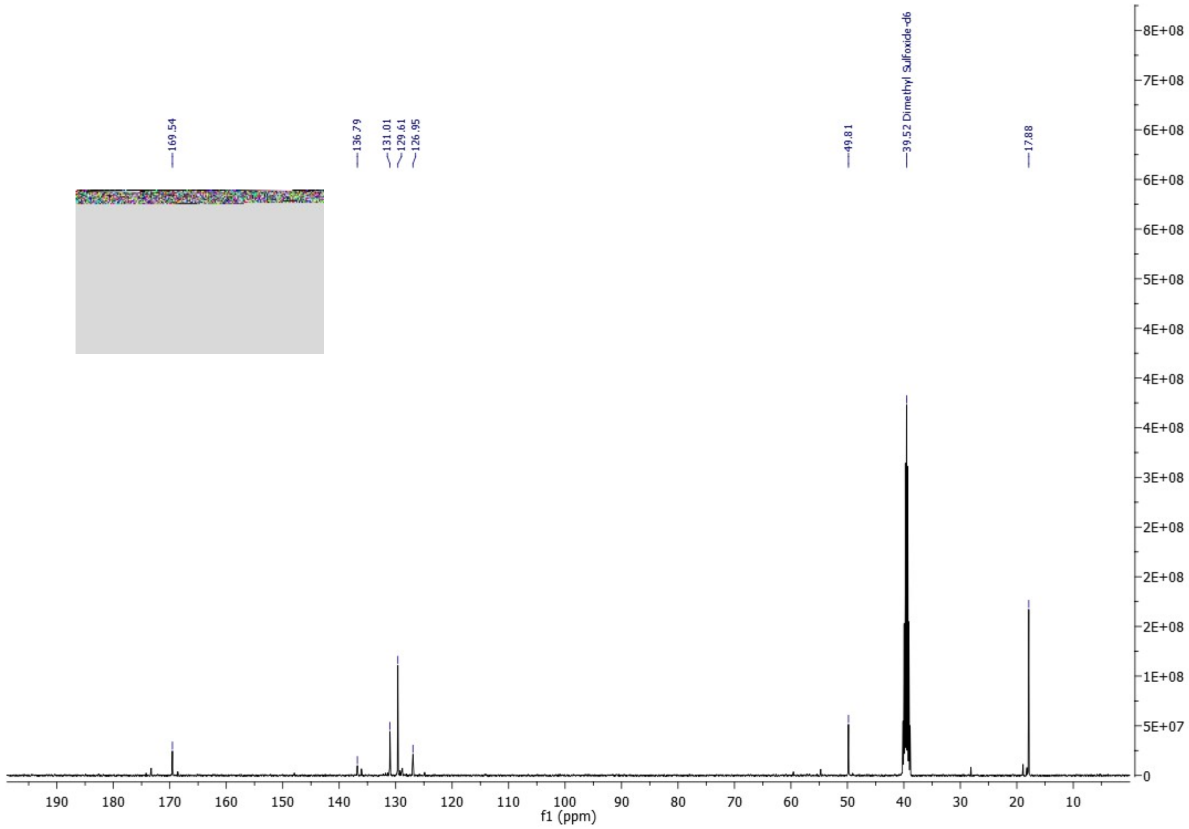

Figure S35: ^{13}C NMR of *N*-(2,6-Dimethylphenyl)glycine (3l)

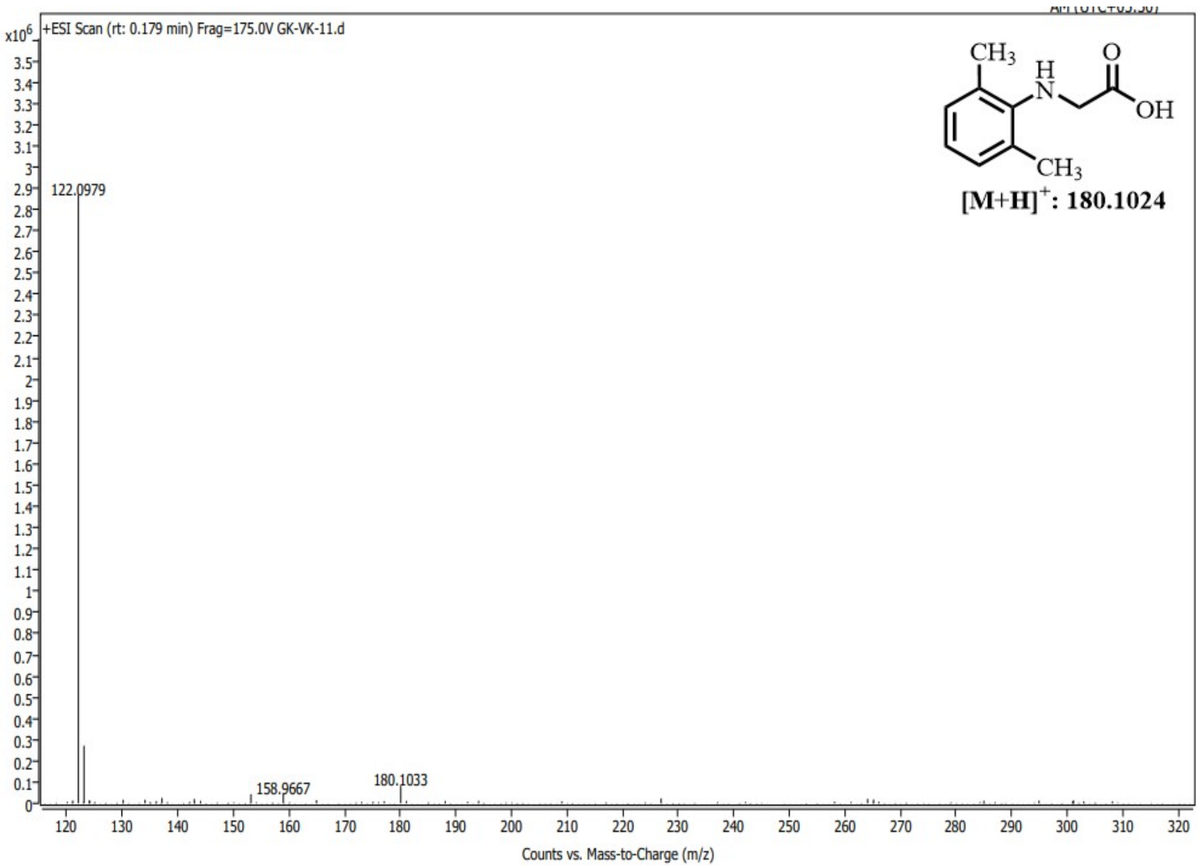

Figure S36: HRMS of *N*-(2,6-Dimethylphenyl)glycine (3l)

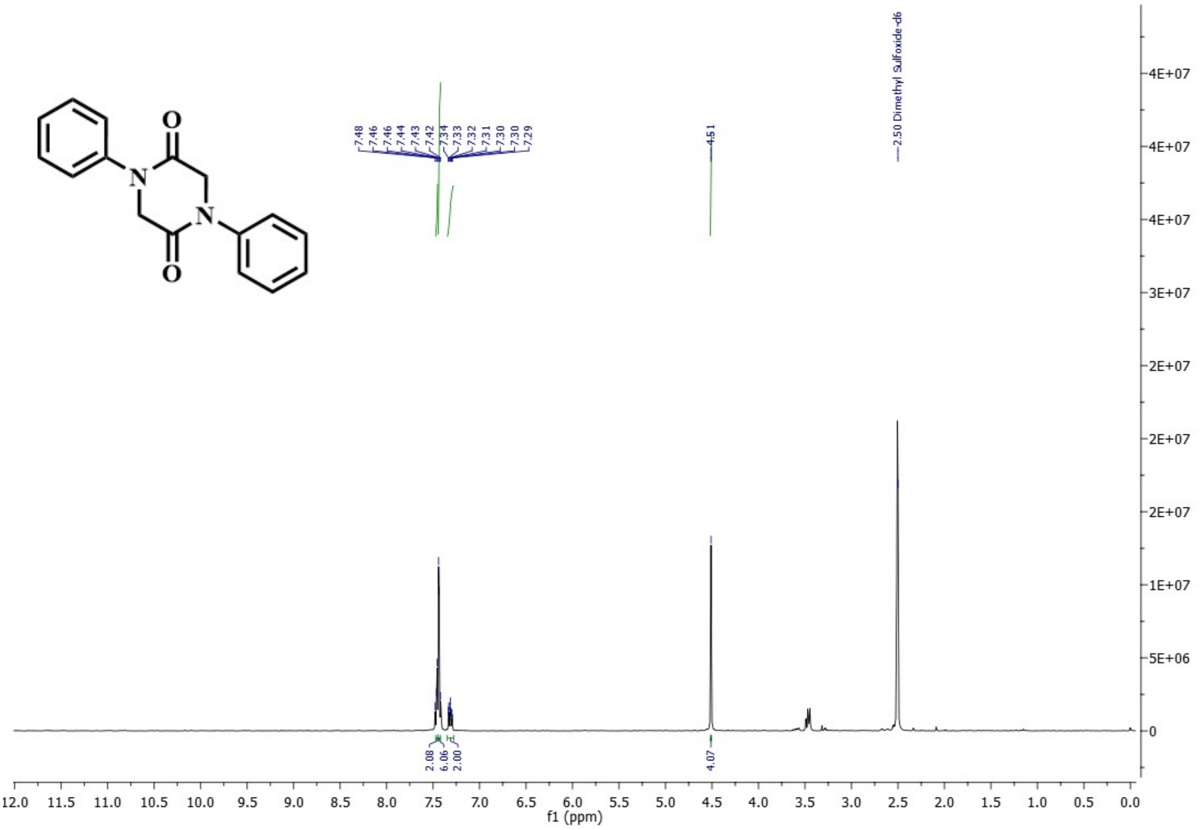

Figure S37: ^{1}H NMR of 1,4-diphenylpiperazine-2,5-dione (2a)

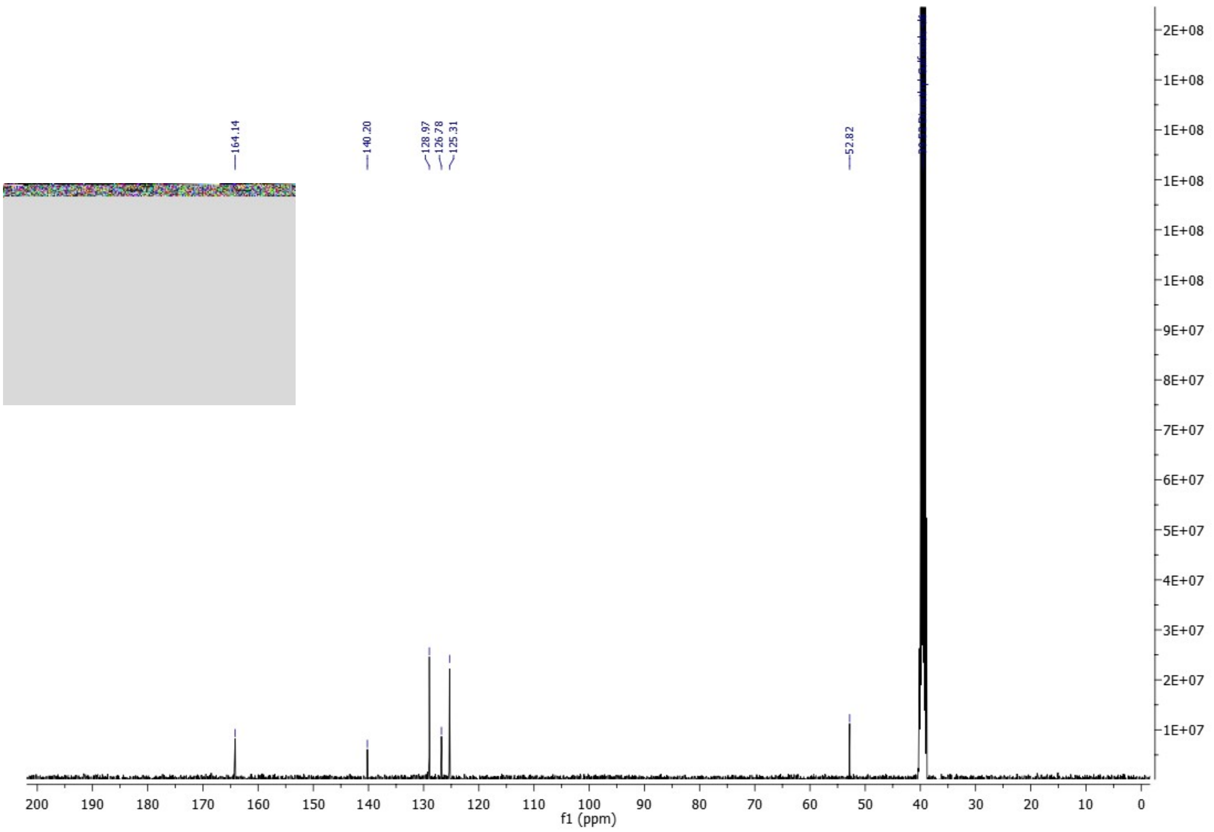

Figure S38: ^{13}C NMR of 1,4-diphenylpiperazine-2,5-dione (2a)
